# Disease-modifying pharmacological treatments of type 1 diabetes: Molecular mechanisms, target checkpoints, and possible combinatorial treatments

**DOI:** 10.1016/j.pharmr.2025.100044

**Published:** 2025-01-23

**Authors:** Liudmila Kosheleva, Daniil Koshelev, Francisco Alejandro Lagunas-Rangel, Shmuel Levit, Alexander Rabinovitch, Helgi B. Schiöth

**Affiliations:** 1 Department of Surgical Sciences, Functional Pharmacology and Neuroscience, Uppsala University, Uppsala, Sweden; 2 Laboratory of Pharmaceutical Pharmacology, Latvian Institute of Organic Synthesis, Riga, Latvia; 3 Diabetes and Metabolism Institute, Assuta Medical Centers, Tel Aviv, Israel; 4 Kelowna, BC, Canada

## Abstract

After a century of extensive scientific investigations, there is still no curative or disease-modifying treatment available that can provide long-lasting remission for patients diagnosed with type 1 diabetes (T1D). Although T1D has historically been regarded as a classic autoimmune disorder targeting and destroying pancreatic islet *β*-cells, significant research has recently demonstrated that *β*-cells themselves also play a substantial role in the disease’s progression, which could explain some of the unfavorable clinical outcomes. We offer a thorough review of scientific and clinical insights pertaining to molecular mechanisms behind pathogenesis and the different therapeutic interventions in T1D covering over 20 possible pharmaceutical intervention treatments. The interventions are categorized as immune therapies, treatments targeting islet endocrine dysfunctions, medications with dual modes of action in immune and islet endocrine cells, and combination treatments with a broader spectrum of activity. We suggest that these collective findings can provide a valuable platform to discover new combinatorial synergies in search of the curative disease-modifying intervention for T1D.

**Significance Statement:**

This research delves into the underlying causes of T1D and identifies critical mechanisms governing *β*-cell function in both healthy and diseased states. Thus, we identify specific pathways that could be manipulated by existing or new pharmacological interventions. These interventions fall into several categories: (1) immunomodifying therapies individually targeting immune cell processes, (2) interventions targeting *β*-cells, (3) compounds that act simultaneously on both immune cell and *β*-cell pathways, and (4) combinations of compounds simultaneously targeting immune and *β*-cell pathways.

## I Introduction

Type 1 diabetes (T1D) is an organ-specific multifactorial autoimmune disease that progressively leads to complete destruction of the insulin-producing pancreatic islet *β*-cells and the need for lifelong insulin therapy. The most desirable and currently unattainable objective of therapeutic intervention is to prevent or halt the initiation and advancement of autoimmunity, counteract the loss of *β*-cells, and restore pancreatic islet endocrine cell functions. Since the discovery of insulin in 1921, significant technological advances in blood glucose monitoring and therapeutic insulin delivery have tremendously improved the quality of life for patients with T1D. Despite this, no cure or long-lasting remission-inducing therapy for T1D has been found, and only a minority of T1D patients are able to maintain optimal glycemic control over the long term (Mayer-Davis et al, 2017; Ashrafzadeh and Hamdy, 2019).

Despite the last 100 years of intensive clinical research, the primary achievement of therapeutic intervention in T1D is predominantly in temporarily delaying the progression of the disease in high-risk populations, mainly by transient interruption of the autoimmune assault on *β*-cells at the onset of T1D (Skyler and Ricordi, 2011). In recent decades, many research groups composed unique observations into different layers and perspectives of the disease, offering a kaleidoscope of distinct insights into specific aspects of T1D pathogenesis. Rorsman and Ashcroft elegantly showed that in order to mitigate severe pancreatic damage associated with different types of diabetes, it is essential to review the *β*-cell electrophysiology and insulin exocytosis (Rorsman and Ashcroft, 2018). Meanwhile, as autoimmunity remains a fundamental aspect of disease manifestation, Bluestone and colleagues provided a comprehensive analysis of immune dysfunction in the emergence and advancement of T1D (Bluestone et al, 2021). They proceeded to evaluate the major existing immune interventions and briefly note the significance of interventions targeting *β*-cells to enhance the efficacy of future treatments. This particular concept is thoroughly examined by Roep and colleagues, who propose intrinsic dual-vulnerability of the immunological and endocrine systems, with *β*-cells acting as an initiator and active contributor to autoimmune assault in T1D (Roep et al, 2021). This “dual-vulnerability hypothesis” is a noteworthy aspect to contemplate, given the limited clinical efficacy reported with several specific immune treatments. The increased efficacy of repurposed drugs that possess mechanistic targets in both *β*-cells and immune cells, such as baricitinib and verapamil (Ovalle et al, 2018; Waibel et al, 2023), further emphasizes the need for a broadly focused pharmacological intervention. We propose that integrating significant research discoveries with clinical outcomes from various therapeutic interventions, and insights from expert reviews can collaboratively advance a new disease-modifying intervention in T1D.

The objective of this study was to provide a comprehensive review of the established pathophysiology of the disease encompassing possible and existing therapeutic interventions. A holistic perspective allows for the identification of synergies and opportunities in the combination of existing treatments, with the ultimate goal of enhancing therapeutic effectiveness. We performed an extensive analysis of the nature of T1D etiology and have identified fundamental mechanisms responsible for *β*-cell function in health and disease. Next, we identified specific pathways that may be targeted by pharmacological interventions using either existing or new experimental pharmaceutical components. Subsequently, we compiled a comprehensive assessment of over 20 possible pharmaceutical intervention treatments, focusing on their respective mechanisms of action. We separated the existing interventions into (1) immune-modifying therapies capable of individually targeting different processes in various immune cells, (2) interventions individually targeting *β*-cells, (3) individual compounds addressing pathways in both immune and *β*-cells simultaneously, and finally, (4) combinations of compounds simultaneously targeting multiple pathways in immune and *β*-cells. This study also aims to provide the foundation for identifying new and combinational strategies that incorporate crucial pharmacological targets in both immune and *β*-cells. Such an approach holds the potential to synergistically enhance the efficacy of current treatment options and provide a comprehensive disease-modifying solution for patients with T1D in the future.

## II Initiation and progression of type 1 diabetes

### A Human leukocyte antigen genetic risk factors associated with type 1 diabetes

A particular target of pharmaceutical intervention of T1D onset is concentrated on dynamic antigen presentation by *β*-cell human leukocyte antigen (HLA) complexes, leading to the initiation and progression of autoimmunity. HLA molecules are frequently divided into 2 distinct groups: HLA class I present on all nucleated cells, and HLA class II, which are mostly expressed on activated T cells, B lymphocytes, and antigen-presenting cells (APCs; Noble and Valdes, 2011). HLA genes encode all cell surface proteins that display antigenic peptides and can be seen and identified by the effector immune cells and trigger an immune response. Like all cells, human islet *β*-cells are well known to express HLA class I molecules, which are significantly upregulated in the early stages of T1D (Richardson et al, 2016) and are recognized by islet-reactive CD8+ T cells (Kronenberg et al, 2012). Some of these autoantigenic peptides develop and progress sequentially as opposed to simultaneously (Yu et al, 1996), and HLA associations, in particular, can determine a person’s predisposition to 1 of 3 T1D subtypes: acute onset, fulminant, or slowly progressive (Kawabata et al, 2009). Importantly, people with a particular T1D genetic susceptibility can experience heterogeneous immune responses to the disease onset, progression, and therapeutic intervention strategies, which was observed on a number of occasions in clinical trials (Schenker et al, 1999; Krischer et al, 2017; Hannelius et al, 2020).

Of note is that until recently, the expression of HLA class II proteins on *β-*cells of T1D-susceptible individuals has been a subject of scientific debate. Forty years ago, Gian Franco Bottazzo identified HLA-DQ genotype responsible for expression of HLA class II cell surface receptors in *β*-cells of a deceased newly onset T1D patient (Bottazzo et al, 1985), and a year later, the term was coined “suicide,” as a mechanism of pancreatic islet *β*-cell destruction in T1D, in addition to autoimmunity (Bottazzo, 1986). This is particularly significant in the pathogenesis of T1D, as it shows *β*-cell ability to directly interact with islet-infiltrating CD4+ T cells (Russell et al, 2019) in an APC-like manner, thereby actively contributing to their demise. In 2008, Erlich and colleagues identified a cluster of 3 genes with a very high risk of T1D, known as a “superlocus” in HLA-DRB1–HLA-DQA1–HLA-DQB1 region responsible for encoding the HLA-DR-DQ proteins for HLA class II expression (Erlich et al, 2008). Since then, variations in the HLA class II genes (chromosome p21.3), in particular, have been identified to account for the majority of genetic risk associated with T1D (Pociot and Lernmark, 2016). By 2015, Hu and colleagues also identified 3 amino acid positions (HLA-DQ*β*1 position 57, HLA-DR*β*1 positions 13 and 71), accounting for 90% of HLA class II phenotypic variance responsible for presenting antigenic peptides that activate autoreactive T cells and drive significant hereditary T1D risk (Hu et al, 2015). It was soon discovered that HLA class II recognized autoantigens are found to be enzymatically modified by *β*-cells undergoing endoplasmic reticulum (ER) stress (Marre et al, 2018). By 2019, HLA-DR-DQ genotype carriers were identified with the highest risk of developing T1D and having upregulated T-cell proliferation response to *β*-cell antigens (Claessens et al, 2020). An elegant study by Quesada-Masachs in 2021 showed that HLA class II proteins were found in 24.31% of insulin-containing islets of T1D donors, almost half of HLA class II signal was colocalized with insulin and 27.5% of these islet cells coreleased HLA class II molecules with insulin (Quesada-Masachs et al, 2022). This shows a definite overlap between autoimmune response and islet metabolic dysfunction, a concept that is not particularly novel, with extensive studies supporting the notion that metabolic stress, dysfunction, and fragility of the *β*-cells need to be addressed concurrently with immune therapies to promote sustainable remission in T1D (Eizirik et al, 2009; Campbell-Thompson et al, 2012; Roep et al, 2021).

### B Other genetic risk factors associated with type 1 diabetes onset

A number of research teams tried to find genetic risk factors for T1D caused by non-HLA loci (Bell et al, 1984; Pugliese et al, 1997; Jerram and Leslie, 2017) and predisposed *β*-cell fragility (Dooley et al, 2016; Liston et al, 2017), particularly connected to ER stress and apoptosis caused and mediated by interferon alfa (Marroqui et al, 2017). Importantly, T1D genetic risk scores have an inverse correlation with age at diagnosis (Redondo and Concannon, 2020) and when examining the relationship between T1D risk loci and patient age at T1D manifestation, Inshaw and colleagues (Inshaw et al, 2018) found that younger children (<7 group compared with the ≥13 group) have a higher prevalence of T1D-associated variations. Notably, in their Supplemental Material, the allele frequency of *β*-cell-associated GLIS3 and CTSH alleles include data on ages up to >35 years with visible increases in allele frequency from 20 to 25 years at the time of diagnosis. In children, genetic variants predisposing most strongly to T1D were identified as 6 HLA haplotypes and 6 non-HLA regions, including *β*-cells specific GLIS3 and CTSH (Inshaw et al, 2020). Further investigation is required to ascertain the relationship between age, stage of diabetes progression, genetic predisposition, and the efficacy of therapeutic interventions.

### C A viral route to type 1 diabetes onset and β-cell damage

Strong evidence suggests that viral triggers play an important part in T1D onset in multiple instances of manifestation. As part of a self-perpetuating cycle, some viral infections can significantly contribute to inflammation, *β*-cells stress, apoptosis, and spreading of epitopes eventually leading to widespread autoimmunity. This correlation is hardly surprising, as *β*-cells exhibit significantly elevated levels of Coxsackie and adenovirus receptor (CAR) expression, which serves as a receptor for enteroviruses. CAR possesses a terminal SIV motif colocalized with zinc transporter protein 8 (ZnT8), prohormone convertase 1/3 (PC1/3) within the mature insulin secretory granules of human islets (Ifie et al, 2018). A systematic review and meta-analysis of a total of 24 publications, amounting to 4448 participants showed a significant correlation between enterovirus infection and T1D autoimmunity (Yeung et al, 2011). In agreement with these findings, enteroviral RNA has been identified in the serum of patients with recent-onset T1D (Schulte et al, 2010), and higher antibody titers Coxsackie B virus was detected in patients within 3 months of disease onset (Gamble et al, 1969). Diabetes virus detection study (DiViD; Krogvold et al, 2014) was the first study to collect a large sample of pancreatic tissue by pancreatic tail resection from 6 live adult patients with recent-onset T1D (24–35 years old). Subsequent research showed that islets of all 6 patients contained enteroviral capsid protein 1 (VP-1) and supported hypothesis on enteroviral infection playing a significant role at T1D manifestation via interferon signaling pathway (Krogvold et al, 2015, 2022). Intriguingly, a combination of antiviral drugs pleconaril and ribavirin administered for 6 months to pediatric patients and adolescents with recent-onset T1D preserved residual *β*-cell function and C-peptide at 12 months (Krogvold et al, 2023). Earlier research also identified the prevalence of VP-1 confined to the *β*-cells in over 60% of recent-onset T1D patients (Richardson et al, 2009). Several nonexclusive mechanisms may induce insulitis and the subsequent loss of *β*-cells following viral infection; however, the exact sequence of events that initiate a virally induced autoimmune reaction that culminates in the development of T1D is still not well elucidated (Op de Beeck and Eizirik, 2016).

### D Interaction of genetic and environmental factors in type 1 diabetes

It remains unclear whether the significant variation in the incidence of T1D worldwide is a result of genetic predisposition, accumulative environmental factors, or both. Several studies imply a geographic association with the disease, noting that second-generation immigrants frequently have an incidence rate of T1D similar to that of the native population (Delli et al, 2010). Söderström and colleagues examined the Swedish population aged 6–25 years, categorized as adoptees, immigrants, second-generation immigrants, and descendants of Swedish-born parents. It was determined that being born in Sweden elevates the risk of T1D in children from low-incidence regions (Söderström et al, 2012). Meanwhile, Ji and colleagues examined whether the incidence of T1D among Swedish second-generation immigrants and adopted children born abroad was similar to that reported in their countries of origin. Their findings indicate that ethnic variations in genetic composition may significantly contribute to the development of T1D (Ji et al, 2010). Delli and colleagues make a significant discovery that somewhat reconciles these conclusions. They found that although second-generation immigrants to Sweden have an increased risk of T1D compared with their country of origin, their disease differs from native Swedes in HLA genetic markers and autoantigen presentation. As such, second-generation immigrants to Sweden T1D patients are predominantly carriers of HLA-DQ2 haplotype, whereas native Swedes were predominantly carrying HLA-DQ8 haplotype (Delli et al, 2010). The findings indicate that exposure to environmental triggers during gestation or early childhood may be a substantial risk factor for T1D, even when genetic markers differ from those of the local population.

Regnell and Lenmark (2017) conducted a comprehensive examination of vulnerability to T1D, considering the complex interactions among genetic predisposition linked to HLA haplotype variants, non-HLA genes, and environmental influences. They propose that concurrently with a worldwide rise in prevalence, the percentage of persons with T1D possessing the high-risk HLA-DR3/4-DQ2/8 genotype has diminished. The most well investigated environmental factor pertains to infectious agents and viral triggers, which are elaborated upon in section A viral route to type 1 diabetes onset and β-cell damage.

## III Pancreatic *β*-cells regulation in health and disease

Intervention therapies for multifaceted conditions, such as T1D, need to account for the complex regulatory mechanisms that govern the functionality and viability of islet cells in both healthy and diseased states. Insulin-like peptides (ILPs) have a long evolutionary history, and a conserved feature among insects is that several ILPs are produced in a set of median neurosecretory cells in the pars intercerebralis of the brain (Nässel and Broeck, 2016). During the process of mammalian evolution, endocrine *β*-cells acquired the ability to secrete insulin from glucose-sensitive neurons. It is worth noting that the RE1-Silencing Transcription factor (REST), also known as Neuron-Restrictive Silencer Factor (NRSF) transcription repressor, which acts as a negative regulator of neuronal fate in non-neuronal cells, is prominently lacking in neurons as well as *α*- and *β*-cells found in the pancreas (Kioussis and Pachnis, 2009; Atouf et al, 1997). This is why pancreatic *β*-cells are able to synthesize inhibitory neurotransmitter *γ*-aminobutyric acid (GABA) in levels comparable with those of GABAergic neurons (Braun et al, 2010) and express many identical neuronal genes and functional surface receptors (Arntfield and van der Kooy, 2011), including type II voltage-dependent sodium channel (Philipson et al, 1993), glutamate signaling system (Maechler and Wollheim, 1999; Cabrera et al, 2008; Otter and Lammert, 2016), GLP-1, and GABA receptors. Some of these neuronal mechanisms in endocrine cells are incremental in T1D-associated metabolic dysfunctions and can present therapeutic targets in alignment with *β*-cell electrical activity regulation.

### A Appropriate β-cell function and regulation of electrical activity

**Fig. 1:**
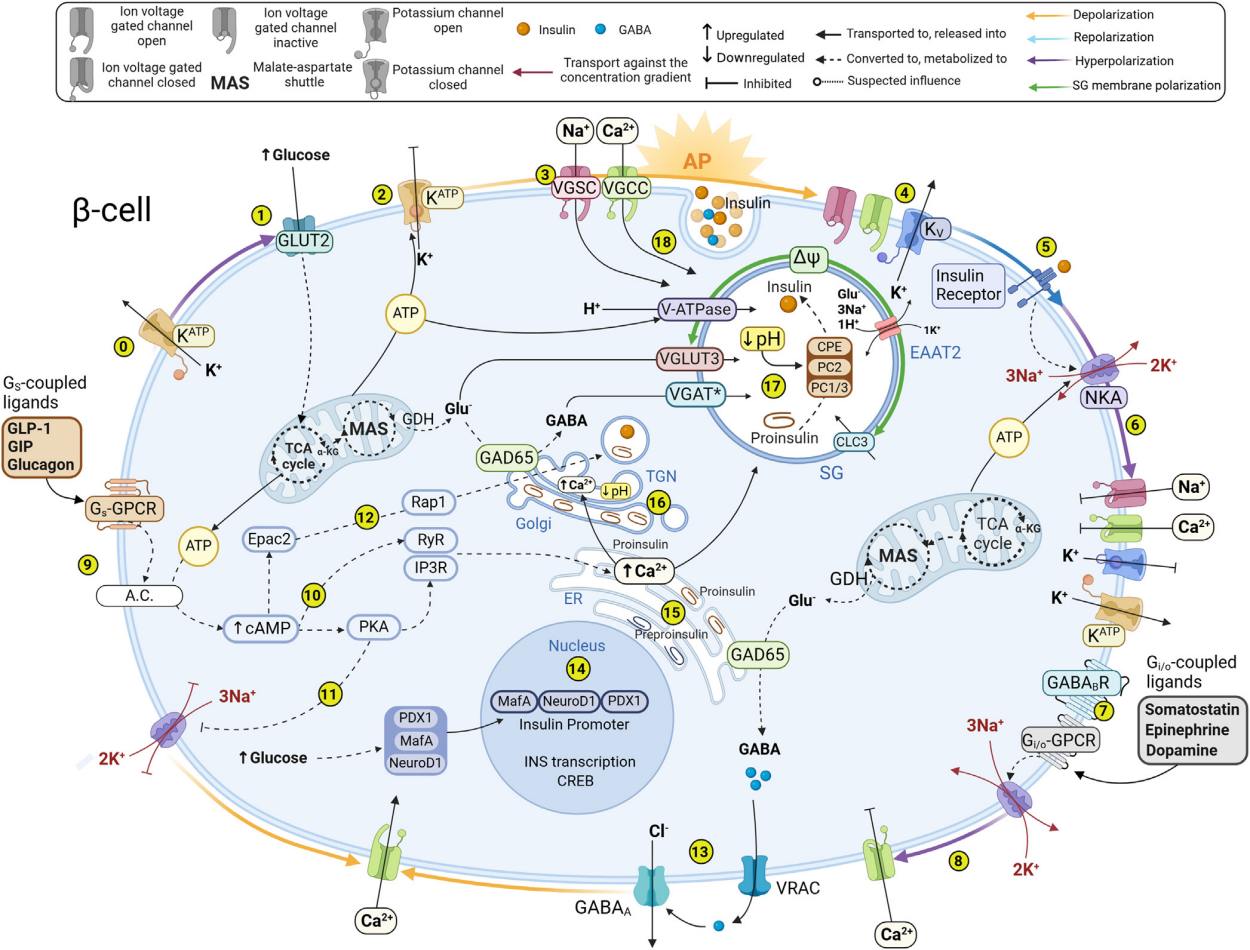
*β*-cell regulation of electrical activity and insulin secretion. *β*-cell function, Ca^2+^ management, and insulin secretion are regulated by the coordinated interaction of ionic movements. (0) In the absence of glucose, the K_ATP_ channel is open, maintaining the *β*-cell membrane hyperpolarized and electrically silent. (1) Glucose influx initiates electrical activity through an increase in metabolically produced ATP, (2) which binds to K_ATP_ and gradually reduces its conductance, causing an increase in the *β*-cell membrane potential. (3) *β*-cell is slowly depolarized in waves, activating voltage-gated calcium (VGCC) and sodium (VGSC) channels and eliciting exocytosis of insulin. (4) Continued depolarization subsequently activates voltage-gated potassium channels (K_V_). (5) K^+^ efflux initiates and regulates the repolarization and consequent hyperpolarization of the cell membrane action potential. ATP-dependent Na^+^/K^+^-ATPase (NKAs) activate by multiple metabolic factors, including insulin receptors and once activated (6) further hyperpolarize *β*-cell membrane, temporarily halting *β*-cell electrical excitability, closing K_V_ and inhibiting influx of Na^+^ and Ca^2+^ entry, thereby reducing insulin secretion. (7) G_i/o_-GPCR ligands signaling also activates NKAs and (8) hyperpolarizes *β*-cell membrane halting Ca^2+^ influx and insulin secretion. Reduction in ATP during hyperpolarization allows a new increase in *β*-cell membrane potential, leading to another wave of depolarization and insulin secretion. (9) The GSIS is potentiated by G_S_-GPCR stimulation with GLP-1, GIP, and other G_S_-ligands that activate adenylyl cyclase (AC) thereby elevating levels of intracellular cAMP. cAMP drives changes including Ca^2+^ influx, mobilization, and the enhanced fusion competence of secretory granules. (10) Elevation of intracellular cAMP concentrations and the activation of cAMP-dependent protein kinase A (PKA) leads to the opening of IP_3_ receptors in ER, releasing Ca^2+^ from internal stores, notably the ER, thereby potentiating GSIS and promoting insulin exocytosis even in Ca^2+^ depleted conditions. (11) PKA activity in *β*-cell will also inhibit NKA function and promote *β*-cell depolarization. (12) Independently of PKA, cAMP also activates Epac2-mediated engagement of Rap1 in *β*-cells that increase the size of the nondocked granule pool and facilitate recruitment and density of the granules to the plasma membrane, thereby potentiating insulin secretion even at low concentrations of glucose. (13) Depolarization independent from glucose influx can occur when *β*-cell is exposed to GABA, through GABA_A_ receptor (GABA_A_R) opening allowing for Cl^-^ efflux. GABA is synthesized in *β*-cells from glutamate with the GAD65 enzyme and can activate GABA receptors on the *β*-cell in a positive feedback loop. GAD65 is distributed between ER/Golgi membrane-anchored, vesicular, and cytosolic localizations. Although a minority of *β*-cells display vesicular GABA colocalization with insulin, most *β*-cells release GABA via volume regulatory anion channels (VRAC) in a pulsatile pattern, occurring in rhythmic bursts independent of glucose concentration. (14) Insulin expression in *β*-cells is achieved by a glucose-dependent transcriptional program. Three transcriptional regulators, PDX1, NeuroD1, and MafA are responsible for glucose-induced insulin gene transcription and *β*-cell-specific function. (15) Insulin gene encodes mRNA preproinsulin, which is translated and translocated across the ER membrane where the signal peptide is removed. The resulting proinsulin molecules are subsequently folded and transported to the Golgi apparatus via the ER-Golgi interface compartment. (16) Upon exposure to acidity and high Ca^2+^ within the Golgi apparatus, the soluble cargo precursor proteins aggregate and bind Ca^2+^, which triggers the aggregation of proinsulin molecules in the trans-Golgi network (TGN). Insulin secretory granule (SG) cargo, including proinsulin, is packaged into granules that bud off from the TGN. (17) SG matures by acidification through the action of the vesicular ATP-dependent proton pump (V-ATPase). Proton (H^+^) transport over the granular membrane results in the development of a considerable membrane potential (ΔΨ) and pH changes. Uptake of negatively charged glutamate (Glu-) by VGLUT3 sets up a counter-charge movement which decreases the granular membrane polarization allowing sustained H^+^ transport by the V-ATPase. EAAT2 in the SG provides a mechanism for the release of accumulated Glu^-^, 3Na^+^, and an H^+^ exchanged with 1K^+^, which decreases the granular membrane polarization allowing sustained acidification by the V-ATPase. Cl^-^ fluxes through the CLC3 channel in the SG membrane provide additional counterconductance for continuous granular acidification. Acidification lowers the pH in the granule and activates prohormone-processing enzymes PC1/3, PC2, and CPE that convert proinsulin to active insulin inside the SG. (18) Insulin exocytosis occurs through a process involving SG docking to the plasma membrane, followed by priming and inward Ca^2+^ and Na^+^ flux-dependent release.

Rorsman and Ashcroft present a thorough and comprehensive review of *β*-cell electrical activity and insulin exocytosis (Rorsman and Ashcroft, 2018). To provide sufficient context for therapeutic pathways of existing pharmaceutical interventions, in our work, we offer a short summary of the main mechanisms involved, which may be found in Fig. 1. In electrically excitable cells, such as neurons and endocrine cells, an action potential is characterized by a rapid sequence of variations in the membrane voltage. Unlike neurons, which experience depolarization as an “all-or-nothing” event, *β*-cells undergo glucose-induced depolarization in the form of slow waves, followed by a plateau phase from which action potentials (AP) are rapidly fired (Jacobson and Philipson, 2007). Elevation of glucose leads to mitochondrial production of intracellular ATP, which binds to the ATP-sensitive potassium channel (K_ATP_) and reduces its activity. The *β*-cell is then slowly depolarized, and its membrane reaches an activation threshold for the opening of voltage-gated calcium (Ca^2+^) and sodium (Na^+^) channels and subsequent insulin release. This process is known as glucose-induced insulin secretion (GSIS). Continued depolarization gradually activates voltage-gated potassium channels (K_V_), leading to potassium efflux-mediated membrane repolarization, followed by hyperpolarization and diminished insulin secretion. Intriguingly, *β*-cells also possess K_ATP_-independent Na^+^/K^+^-ATPase (NKA)-mediated hyperpolarization mechanisms activated by G_i/o_-GPCRs signaling in response to somatostatin, epinephrine, or dopamine (Dickerson et al, 2022). These processes demonstrate a major electric regulatory network within the islet and paracrine signaling system that controls the handling of calcium in *β*-cells and the production of insulin in the presence of other hormonal stimuli.

In addition to K_ATP_ inhibition by means of ATP production, mitochondrial metabolism also generates essential distinct mitochondrial messenger—neurotransmitter glutamate, necessary for insulin exocytosis (Maechler and Wollheim, 1999), GABA synthesis (Menegaz et al, 2019), and *β*-cell regulation (Otter and Lammert, 2016). Glutamate N-Methyl-D-Aspartate receptors (NMDARs) activate by binding both glycine and glutamate, with the concomitant depolarization of the cell membrane (Hardingham and Bading, 2010). NMDAR opening initiates an influx of calcium and sodium, efflux of potassium ions in addition to the extrusion of magnesium ions from the channel pore activity. NMDAR signaling reactivates K_ATP_ potassium channels, repolarizes cell membranes, and inhibits GSIS (Wollheim and Maechler, 2015). Importantly, in a healthy *β*-cell, some intracellular glutamate is synthesized to GABA by glutamic acid decarboxylase 65 (GAD65), which is distributed in the cytosol, anchored to the Golgi network and other vesicle membranes (Kanaani et al, 2008). Physiologically, GABA functions as a fundamental paracrine and autocrine signaling molecule, regulating the electrical activity and function of endocrine cells. In the pancreas, GABA can adjust electrical activity in endocrine cells to target the excessive production of glucagon, which has a substantial impact on the development of diabetic hyperglucagonemia (Rorsman et al, 1989; Braun et al, 2004). Subsequent sections of this review present a comprehensive analysis of pharmacological treatments that specifically target GABA-mediated signaling.

### B Intervention checkpoint: induction of β-cell apoptosis by hyperglycemia

#### 1 TxNIP assembly and transcription

**Fig. 2:**
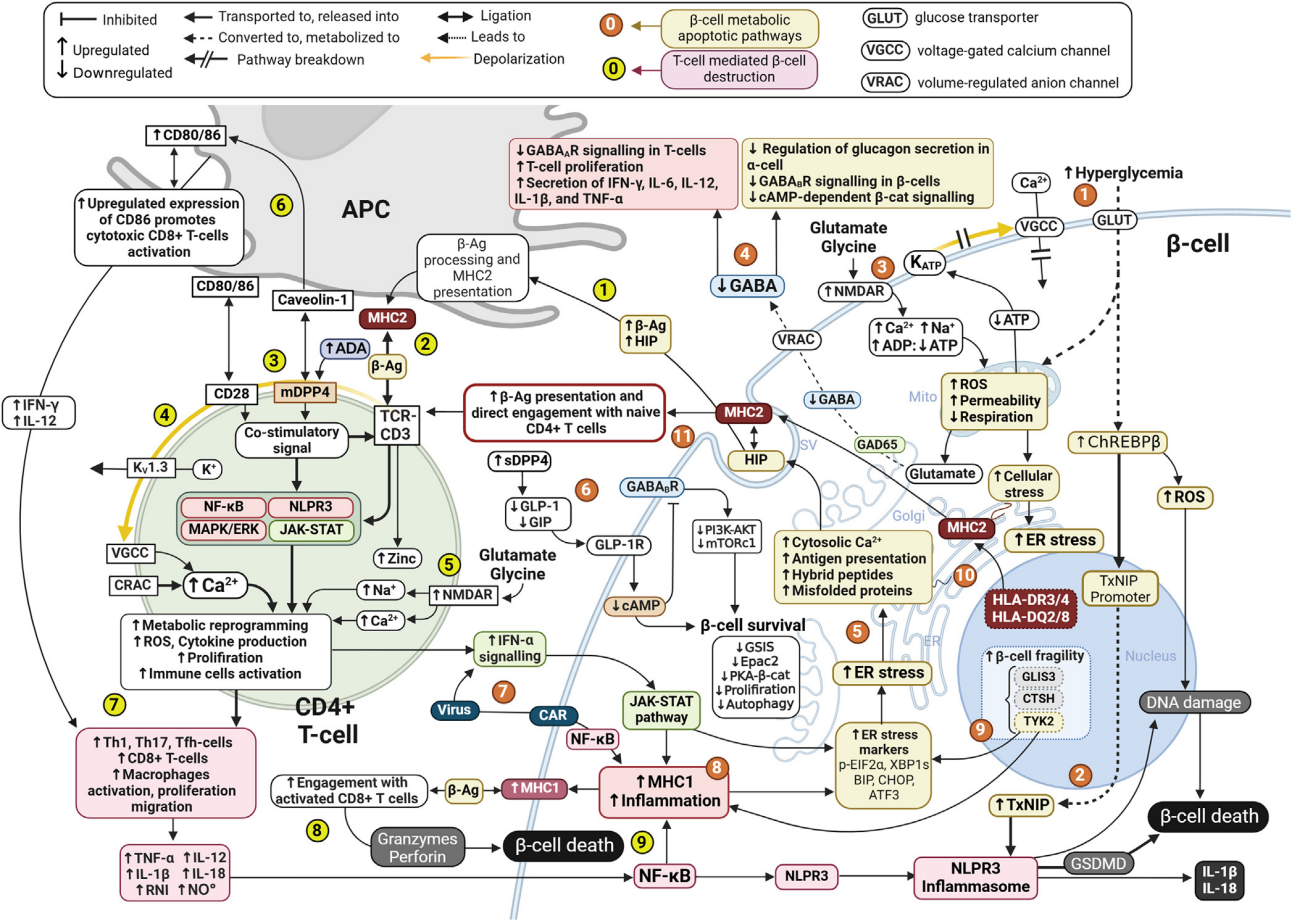
Proposed chart of T1D dual vulnerability initiated by *β*-cell metabolic dysfunction and CD4+ T-cell activation. *β*-cell metabolic dysfunction processes (orange numbers). (1) Chronic exposure to hyperglycemia causes elevation of the carbohydrate response-element binding protein *β* (ChREBP*β*) and Ca2+ influx, leading to increased transcription of proapoptotic TxNIP. (2) In the *β*-cell TxNIP upregulates transcription of NLRP3 inflammasome, a protein complex responsible for caspase-1–dependent maturation of the proinflammatory cytokines IL-1*β* and IL-18 and gasdermin D(GSDMD)-mediated apoptotic cell death. (3) The onset of metabolic dysfunction may also arise from the impaired glutamate transmission and dysregulation of the *β*-cell’s N-methyl-d-aspartate receptors (NMDARs), which play a crucial role in controlling insulin secretion, electrical activity, and cell survival by modulating the influx of calcium ions (Ca2+) and sodium ions (Na+). Increases in cytosolic Ca2+ levels cause increased permeability of mitochondria, altered mitochondrial respiration, the release of reactive oxygen species (ROS), and activation of other proapoptotic factors. Breakdown of the depolarization mechanisms precludes repolarization via the opening of voltage-gated K+ channels (KV), which can have an excitotoxic effect on *β*-cells and increase intracellular Ca2+ levels. (4) *β*-cell metabolic dysfunction could suppress GABA synthesis from intracellular glutamate by glutamic acid decarboxylase (GAD65) and release of GABA from *β*-cells. GAD65 is one of the major target antigens in T1D, and GAD65 autoantibody is a diagnostic marker for T1D. Patients with T1D exhibit a significant reduction in plasma GABA levels. This impacts islet regulatory pathways, including a key *β*-cell mechanism for glucagon regulation in the *α*-cells via chloride (Cl-) influx through GABAAR and hyperpolarization of the *α*-cell plasma membrane. Insufficient extracellular GABA levels also hinder cAMP-dependent *β*-cell survival pathways, such as *β*-catenin-mediated (*β*-cat) signaling and GABAB receptor (GABABR) initiated PI3K-AKT-cascades. Additionally, reduced GABA signaling will also fail to inhibit T cell proliferation through activating GABAA Cl- channels and reducing the secretion of interferon gamma, IL-6, IL-12, IL-1*β*, and TNF-*α*. (5) Cellular stress includes endoplasmic reticulum (ER) stress which triggers an unfolded protein response (UPR) consisting of impaired RNA transcription and translation, leading to depletion of ER Ca2+ stores and accumulation and release of misfolded proteins, hybrid insulin peptides (HIP) and increased autoantigen presentation. (6) Elevated serum DPP-4 (sDPP-4) levels are found to be elevated in T1D patients and degrade incretins such as GLP-1, thereby preventing incretin-induced cAMP downstream signaling in *β*-cells, which interferes with GSIS and impairs cAMP-dependent cell survival processes. (7) An inflammatory trigger event in *β*-cells (eg, viral infection via coxsackie and adenovirus receptor [CAR]) would initiate secretion of type I interferons (interferon-*α*/*β*) by the immune system or other cells, leading to activation of the JAK-STAT pathway and the NF-*κ*B pathway and increase of ER stress in the *β*-cell. (8) interferon alfa has been identified as a key driver of increased expression of HLA class I molecules of the major histocompatibility complex I (MHC1) system on *β*-cells in the early stages of T1D. These MHC1 molecules bind *β*-cell–derived autoantigens (*β*-Ag) and activate CD8+ T-cells, as well as upregulate ER stress sensors and markers in the *β*-cell (eg, p-EIF2*α*, XBP1s, BIP, C/EBP homologous protein (CHOP), ATF3, and ATF6). (9) The T1D-associated gene TYK2 contributes to the activation of interferon alfa-mediated MHC1 expression in *β*-cells. (10) HLA-DR3/4 and HLA-DQ2/8 haplotypes of the HLA class II molecules of the MHC2 system are those associated with T1D. (11) Expression of MHC2 molecules by islet *β*-cells is an aberrant feature that confers *β*-cells the ability to bind *β*-Ag associated with T1D and engage CD4+ T-cells in an antigen-presenting cell (APC)-like manner, initiating autoimmunity. 2. Autoimmune processes (yellow numbers). Immune cell responses against antigens and *β*-cell–derived *β*-Ag play a central role in the pathogenesis of T1D. (1) Independently of MHC1 and MHC2 *β*-Ag presentation, *β*-cells under metabolic stress continuously secrete fused peptide fragments or hybrid insulin peptides (HIPs) that are recognized by APCs as autoantigen targets for pathogenic islet-infiltrating T-cells. (2) Some *β*-Ag are processed by APCs and presented to naïve CD4+ T-cells as antigens by MHC2 molecules on the surface of the APC. (3) CD4+ T-cell activation and differentiation depends on the signal strength received by the T-cell receptor (TCR) via the binding of MHC2 and on costimulation signals. CD28 and membrane-bound DPP4 (mDPP4) are prominent co-stimulatory molecules controlling the activation and behavior of naïve CD4+ T-cells. The mDPP4-mediated signal can be co-stimulated with APC in a caveolin-1-mDPP4-CD45-CD3-dependent manner activating the MAPK/ERK signaling pathway, or by the adenosine deaminase (ADA)-mDPP4 pathway activating proinflammatory NF-*κ*B transcription factors. (4) CD4+ T-cells depend on the membrane action potential that is initiated by TCR stimulation for activation, intracellular Ca2+ homeostasis, cytokine production, and proliferation. Autoantigen-activated MHC2-TCR complex depolarizes the T-cell membrane and opens KV1.3 channels that are part of a signaling complex with P56lck (LCK), previously associated with impaired T-cell activation in T1D. K+ efflux opens Ca2+ release-activated channels (CRAC), resulting in significant Ca2+ influx and opening of KCA3.1 channels, thereby sustaining prolonged Ca2+ entry that is needed for further T-cell activation, cytokine release, and proliferation. (5) Upon TCR-activation and metabolic reprogramming, CD4+ T-cells also upregulate the expression of NMDAR, which are glutamate-activated and have an effect on cytokine production, T-cell proliferation, and differentiation. (6) In APCs, mDPP4-caveolin-1 interaction upregulates the expression of CD86 and subsequent engagement and recruitment of CD8+ cytotoxic T-cells, and cytokine secretion. The production by the APCs of cytokines such as IL-12 and interferon gamma promotes and accelerates further differentiation of the CD4+ T-cells into Th1-type cells and inhibits CD4+ Th2 cell production of IL-4 and IL-10. Additionally, interferon gamma upregulates the expression of NADPH oxidase/NOX family proteins that transport electrons from nicotinamide adenine dinucleotide and generate cytoplasmic reactive oxygen species (ROS). (7) Metabolic reprogramming of naïve CD4+ T-cells also leads to an increase in ROS and accelerated proliferation and activation of specialized immune cell subtypes, such as Th1, Th7, and Tfh. Activated CD4+ and CD8+ T-cells also secrete interferon gamma, IL-12, IL-18, and proinflammatory TNF-*α* and IL-1*β*, activating macrophages and stimulating the production of reactive nitrogen intermediates (RNIs). (8) Binding of CD8+ T-cells with *β*-cells, via a T-cell receptor (TCR)-MHC1 complex induces *β*-cell death through secretion of toxic molecules, such as perforin and granzymes. T-cells and macrophages can also destroy *β*-cells by secreting nitric oxide (NO°) and cytotoxic cytokines, subsequently activating a NF-*κ*B signaling profile. Importantly, secretion of these cytokines by CD4+ T-cells may also increase expression of MHC1 on *β*-cells and promote direct assaults by CD8+ T-cells. (9) Autoimmune assaults on the *β*-cells by ROS, RNIs, actions of cytokines, and granzymes that activate caspase enzymes, lead to *β*-cell apoptosis and/or necrosis.

Hyperglycemia plays a major role in *β*-cell loss, but the pathways leading to destructive effects of glucotoxicity remain unclear. Thioredoxin-interacting protein (TxNIP) was recently established as a novel proapoptotic gene in the *β*-cells that is significantly overexpressed during hyperglycemia in T1D (Minn et al, 2005), and its expression in *β*-cells is directly elevated due to the toxic effects of continuous exposure to elevated glucose (Fig. 2). TxNIP binds to the antioxidant enzyme thioredoxin (Txn) and inhibits it, consequently facilitating oxidative damage in *β*-cells and is recognized as one of the key therapeutic targets of cell stress (Choi and Park, 2023) responsible for activating NLRP3 inflammasome (Qayyum et al, 2021). In pancreatic *β*-cells, glucose-stimulated TxNIP transcription is mediated by a glucose-sensitive transcription factor, carbohydrate response-element binding protein (ChREBP; Shalev, 2014). Two major isoforms of ChREBP, *α* and *β*, can have opposing metabolic effects in regulating *β*-cell function. Although CHREBP*α* can have protective effects during oxidative stress, CHREBP*β*, in particular, is the predominant isoform highly elevated in conditions of hyperglycemia and diabetes, leading to *β*-cell death (Katz et al, 2022). One way to prevent TxNIP elevation in the *β*-cell is to target the nuclear-cytoplasmic shuttling of ChREBP and binding to DNA, which is regulated by PKA- and AMPK-mediated phosphorylations. AMPK is the central regulator of cell energy homeostasis, and upon activation, it phosphorylates several downstream effectors responsible for glucose metabolism and cell longevity. Metformin in particular was shown to transiently inhibit complex I of the electron transport chain in the mitochondrion, leading to activation of LKB1-mediated AMP-activated protein kinase (AMPK; Mihaylova and Shaw, 2011). Metformin-activated AMPK directly hinders hyperglycemia-stimulated nuclear translocation of ChREBP and forkhead box O1 (FOXO1), preventing their subsequent recruitment on TxNIP promoter (Li et al, 2015).

#### 2 Endoplasmic reticulum stress and unfolded protein response

Among other hyperglycemia-induced impairment pathways (Marré and Piganelli, 2017; Thomaidou et al, 2020), key contributors of *β*-cell dysfunction in T1D are endoplasmic reticulum (ER) stress and the aberrant unfolded protein response (UPR; Roep et al, 2021; Sahin et al, 2021). The ER contains the largest calcium reserve in the cell and is responsible for maintaining a variety of translation chaperones and enzymes that facilitate protein folding, maturation, and other posttranslational modifications described in detail elsewhere (Anken and Braakman, 2005). Under stimuli caused by transient stressors, *β*-cells activate UPR’s function and promote cell survival. However, exposure to prolonged or chronic stress, commonly associated with diabetic autoimmune and metabolic dysfunction, UPR adaptive response is not sufficient and leads to direct *β*-cell apoptosis and premature loss of *β*-cell mass. In line with this concept, the UPR is commonly associated with the regulation of *β*-cell function, generation of antigenic peptides, and development of T1D (Fig. 2) (Thomaidou et al, 2018).

#### 3 Glutamate-induced β-cell damage

During diabetes, chronic exposure to hyperglycemia leads to glucotoxicity through rapid matrix acidification of the *β*-cells and control of the membrane potential of *β*-cells by affecting the ratio of intracellular ATP/ADP concentrations, elevation of intracellular glutamate, and acceleration of *β*-cell damage via NMDAR (Huang et al, 2017). Extended glutamate stimulation of NMDARs elevates intracellular mitochondrial accumulation of calcium, thereby laying the groundwork for ER dysfunction and oxidative stress (Fig. 2) (Mohsin et al, 2020), eventually culminating in *β*-cell apoptosis (Feissner et al, 2009). Considering the electrical activity of the *β*-cells in disease led Sterk and colleagues to discover that inhibition of NMDARs in human islets can improve the survival of endocrine cells and restore GSIS (Šterk et al, 2021). Marquard and colleagues presented a further series of genetic and pharmacological experiments and found that inhibiting NMDARs, particularly with dextromethorphan (DXM), can significantly improve GSIS through prolonging depolarized state and plateau phase of calcium oscillations (Marquard et al, 2015). These findings were later supported in non-obese diabetic (NOD) mice as a model for T1D, highlighting DXM as a novel safe intervention candidate to be used as adjunct treatment of preclinical (stage 2) or recent-onset T1D (stage 3; Wörmeyer et al, 2024).

### C Intervention checkpoint: existing β-cell stress rescue mechanisms

#### 1 Incretin rescue of GSIS

**Fig. 3:**
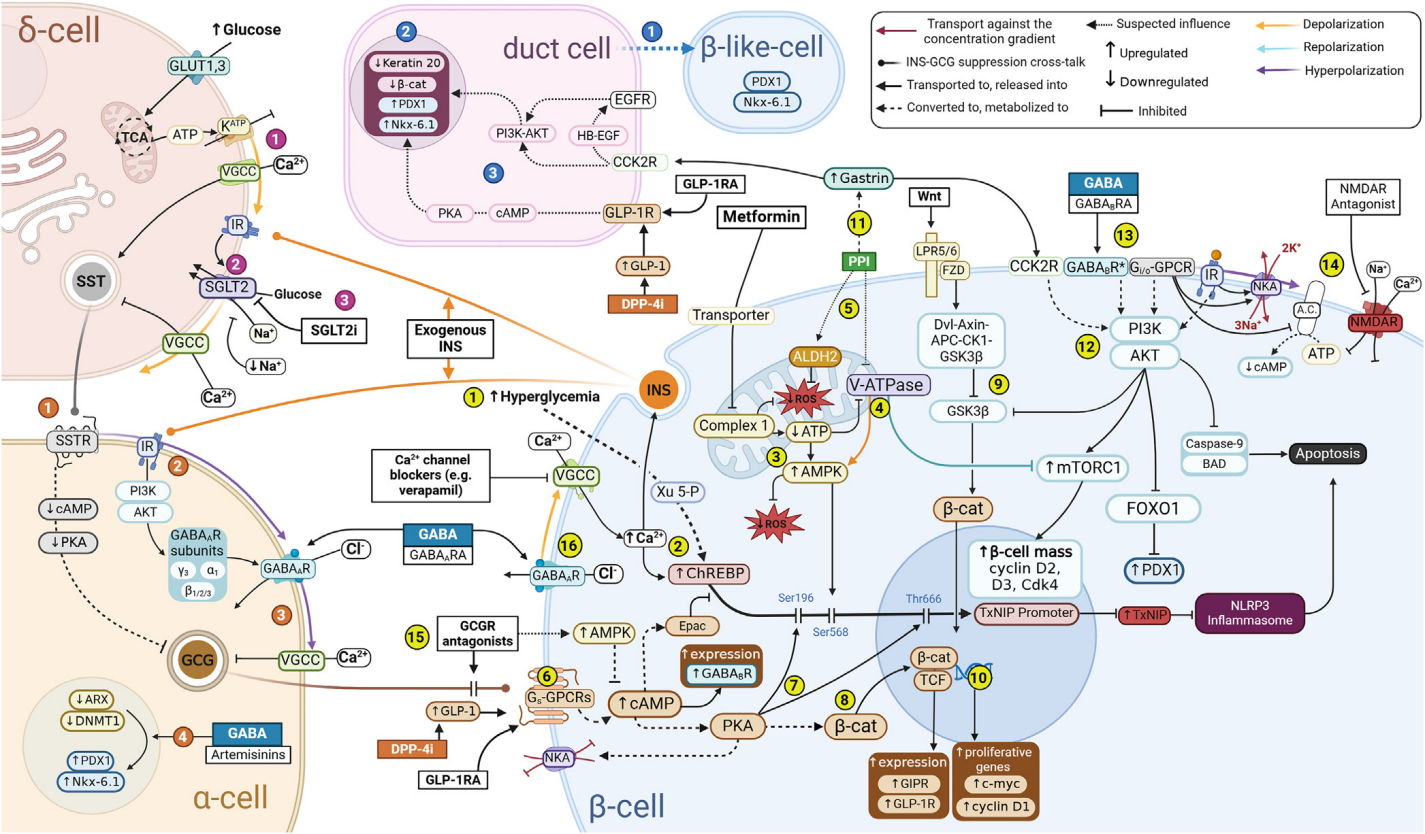
T1D targets for therapeutic interventions in endocrine cells. *β-cell:* This figure illustrates the key targets for pharmaceutical intervention of oxidative stress, glucotoxicity, and apoptosis in pancreatic *β*-cells. (1) Thioredoxin-interacting protein (TxNIP) has an important role and the transcription is activated by carbohydrate-response element-binding protein (ChREBP) which binds to the TxNIP promotor and mediates glucose-induced TxNIP expression. During hyperglycemia, glucose is converted to Xu-5-P, with elevation of cytosolic Ca^2+^ ChREBP is translocated into the nucleus and translated into TxNIP. (2) First-generation Ca^2+^ blockers (diltiazem or verapamil) significantly reduce endogenous TxNIP mRNA expression due to the reduction of cytosolic Ca^2+^. The nuclear-cytoplasmic shuttling of ChREBP and binding to DNA are regulated by PKA- and AMPK-mediated phosphorylations. Metformin transiently inhibits complex I of the electron transport chain in the mitochondrion, leading to inhibition of V-ATPase and LKB1-mediated AMPK activation. Activated AMPK directly hinders ChREBP at Ser568, prevents cytosol-to-nuclear translocation, and inactivates its DNA-binding activity. (4) V-ATPase functions as a sensor switch between AMPK-induced catabolic metabolism and mTORC1 anabolic pathways. Activation of mTORC1 is a major increase of beta-cell mass by modulation of cyclin D2, D3, and Cdk4 activity. (5) Some PPI can also inhibit V-ATPase-Ragulator and induce AMPK-mediated response to ROS. Additionally, omeprazole preserves ALDH2 in mitochondria. Activation of ALDH2 in *β*-cells prevents apoptosis, enhances GSIS, and reduces both the mitochondrial and intracellular ROS levels. (6) Pharmaceutical interventions with GLP-1RA, GCGR mAbs, and DPP-4i-induced GLP-1 elevation stimulate GS-coupled ligands and initiate cAMP-PKA and cAMP-Epac signaling, thereby downregulating TxNIP expression levels in hyperglycemic conditions. (7) PKA phosphorylates ChREBP at Ser196, which inhibits nuclear import at Thr666, which inhibits DNA-binding activity. Downregulation of TxNIP expression reduces caspase-1 expression and prevents caspase-induced apoptosis. (8) *β*-catenin (*β*-Cat) is another pharmaceutical target that can promote proliferation, survival, and function of *β*-cells in diabetic conditions. GLP-1RA activation leads to *β*-Cat stabilization through PKA-mediated phosphorylation. (9) Additionally, Wnt-*β*-cat and PI3K-AKT-mTORC1 pathways share a common inhibition target GSK3*β* and independently can upregulate free cytosolic *β*-cat. (10) In the nucleus *β*-Cat forms the bipartite transcription factor *β*-cat/TCF with a TCF family member (eg, TCF7L2) upregulating GLP-1R and GIPR expression and several proliferative genes, including *c-myc* and *cyclin D1*. (11) PPI indirectly elevates serum gastrin. (12) Stimulation of G-coupled receptors, including gastrin receptor CCK2R, GABABR, G_i/o_-GPCR, and insulin receptors activate PI3K-AKT pathway leading to activation of mTORC1 and inhibition/mediation of GSK3*β*, caspase-9 and Bcl-2-associated death promoter (Bad). (13) Expression of functional GABA_B_R in human islets is restricted and tightly regulated by elevation in cAMP signaling. GABA_B_R is primarily coupled to the G_i/o_-GCGR and their activation leads to hyperpolarization induced closure of VGCC and insulin secretion. (14) NMDAR inhibition with dextromethorphan (DXM) significantly prolongs *β*-cell depolarization state promoting insulin secretion. (15) GCGR mAbs, such as IgG2 mAb volagidemab, bind to the human GCGR, and competitively block GCGR interaction with glucagon, thereby reducing cAMP and elevating AMPK. GCGR antagonism can improve glycemic control but cause adverse events in patients. (16) In β-cells activation of GABA_A_Rs with GABA, benzodiazepines, barbiturates, neurosteroids, and ethanol leads to Cl^-^ efflux and depolarization, opening of VGSC, VGCC, and promoting insulin release. *α-cell:* (1) Insulin inhibits glucagon secretion via insulin-dependent SGLT-2-induced stimulation of somatostatin release by *δ* cells, which downregulate cAMP, activate Gi/o-GPCR and hyperpolarize *α*-cell membrane. (2) Additionally, *β*-cells secretion of insulin and GABA inhibit glucagon secretion via IR activation of PI3K-AKT-dependent membrane assembly and activation of GABA_A_R activity. (3) GABA_A_R activation with GABA leads to Cl^-^ influx, *α*-cell membrane hyperpolarization, and suppression of glucagon secretion. (4) GABA and artemisinins can potentially reprogram *α*-cell into *β*-like-cell by repression or expression of key transcription factors. *δ-cell:* Somatostatin is a paracrine inhibitor of both insulin and glucagon. (1) The δ-cells are electrically excitable, and somatostatin secretion depends on SGLT2 activation by (2) insulin and K_ATP_-induced depolarization, provided there are extracellular Na^+^ and elevated glucose levels. (3) SGLT2 inhibitors were seen to improve clinical parameters in T1D patients but can provoke euglycemic ketoacidosis and increase hepatic glucose production. *Pancreatic exocrine cells*: (1) Gastrin enhances ductal cell transdifferentiation into insulin-producing *β*-like cells. In ductal cells, concurrent administration of gastrin with epidermal growth factor or GLP-1RA has been shown to increase the *β*-cell mass and/or to improve glucose tolerance. (2) Exocrine cells are reprogrammed by upregulating gene expression of the endocrine progenitor markers including PDX1, and Nkx-6.1, and downregulating KRT20 expression present in mature duct cells. (3) In acinar cells, the antiapoptotic action of gastrin is mediated by PI3K-AKT, ERK, and MAPK signaling. The exact mechanism for transdifferentiation of pancreatic exocrine cells into beta-like cells has not been elucidated, but molecular interactions can include gastrin-induced upregulation of heparin-binding epidermal growth factor-like (HB-EGF), transactivation of EGFR, PI3K-AKT-mTORC1, and GLP-1-cAMP-PKA signaling cascades.

Binding of incretins such as glucose-dependent insulinotropic polypeptide (GIP) and glucagon-like peptide-1 (GLP-1) initiates cyclic adenosine monophosphate (cAMP) downstream signaling, which is recognized as the most significant potentiator of insulin secretion (Fig. 3). Canonical GLP-1R signaling initiates the activation of adenylate cyclase (AC) and rapid production of cAMP, which potentiates GSIS by 2 distinct protein-kinase A (PKA)-dependent and -independent mechanisms (Seino et al, 2009). Activated PKA downstream signaling is responsible for many processes related to calcium regulation, such as phosphorylating Kir6.2 and SUR1 K_ATP_ channel subunits (Béguin et al, 1999), stimulation of L-type voltage-gated calcium channels (Bünemann et al, 1999), and calcium release from the intracellular calcium stores such as the ER. PKA-independent mechanism potentiating insulin secretion occurs by cAMP-initiated activation of cAMP-binding protein Epac2 (Epac2), which was recently discovered to regulate the insulin granule density by engaging Ras-like small GTPase Rap1 (Fig. 1) (Shibasaki et al, 2007). These pathways are especially important, as a rescue mechanism for the endocrine system during hyperglycemic conditions, as GLP-1R signaling initiates several metabolic mechanisms that can not only have a short-term effect on *β*-cell function, such as potentiation of GSIS, but also regulate long-term effects, such as *β*-cell survival and proliferation. Potentiating GSIS via GLP-1R-induced signaling can play a crucial role in rescuing the endocrine system short term during hyperglycemic conditions. However, incretin-induced signaling can also have long-term effects, such as regulating the survival and growth of *β*-cells.

#### 2 Autophagic pathway for cell survival

Autophagy is a catabolic cellular process for recycling damaged molecules and subcellular elements via lysosome-mediated degradation. It is fundamental for appropriate *β*-cell function and survival during stressful conditions, including ER stress and pathogenic activity of lysosomal cathepsin proteases, leading to the assembly of highly antigenic epitopes for CD4+ T cells (Reed et al, 2021; Crawford et al, 2022). Many groups hypothesized that defective autophagy and lysosome defects are associated T1D pathogenesis and can directly affect *β*-cell presentation and expression of MHC class I and class II molecules (Russell et al, 2019) in *β*-cells (Fierabracci, 2014; Muralidharan et al, 2021; Mohammadi-Motlagh et al, 2023). As a therapeutic target, impaired autophagy can be the driving force behind general *β*-cell metabolic dysfunction in T1D (Muralidharan et al, 2021), especially considering the importance of lysosomes’ role in the cellular response to stress (Lawrence and Zoncu, 2019). Pharmaceutical regulation of the mTORC1 pathway signaling was found to regulate autophagy during metabolic stress in *β*-cells and can present a specific addressable target in T1D (Bartolomé et al, 2014; Blandino-Rosano et al, 2017; Pasquier et al, 2019). As such, GLP-1R-cAMP-induced signaling has a significant effect in regulating *β*-cell autophagy (Kanasaki et al, 2019). There are numerous reports on how GLP-1R agonist (GLP-1RA) can restore lysosomal function, relieve the impairment in autophagic flux, and promote *β*-cell proliferation and autophagy specifically under metabolic stress (Fig. 2) (Wang et al, 2015a; Zummo et al, 2017; Arden, 2018). Limited data are available regarding DPP-4 inhibitor (DPP-4i) effect on stimulating autophagy in pancreatic cells (Zheng et al, 2018).

#### 3 Wnt/β-catenin pathway-induced β-cell proliferation and survival

Wnt-*β*-catenin pathway in the *β*-cell is responsible for a broad spectrum of processes, including *β*-cell vitality and proliferation, GSIS, and function and secretion of incretin hormones (Napolitano et al, 2023). In pancreatic *β*-cells, Wnt-*β*-catenin pathway is activated by Wnt frizzled receptors and phosphatidylinositol 3-kinase (PI3K)-AKT-mTORC1 downstream signaling. Both cascades lead to the inhibition of glycogen synthase kinase 3*β* (GSK3*β*) and the accumulation of free cytosolic *β*-catenin (Ju et al, 2006; Liu and Habener, 2008). Free *β*-catenin moves from cytosol into the nucleus and combines with T-cell factor (TCF) family of transcription factors, such as TCF7L2, thereby activating the expression of Wnt signaling target *β*-cell proliferative genes, including cyclin D1 and *c-myc* (Fig. 3). A noncanonical cAMP-PKA-mediated phosphorylation of *β*-catenin Ser-675 independently of GSK3*β* was reported by a number of research groups in different cell types by activated protein kinases (eg, P21-activated protein kinase), insulin, GLP-1, and IGF-1 (Hino et al, 2005; Chiang et al, 2013; Chiang and Jin, 2014).

In particular, Liu and colleagues elucidated a mechanism in pancreatic cells by which GLP-1RA-mediated activation of cAMP-PKA phosphorylates *β*-catenin on Ser-675 and thereby enhances the expression of TCF7L2 (Liu and Habener, 2008). More recently, GLP-1RA-induced noncanonical *β*-catenin pathway of Ser-675 phosphorylation was observed in combination with GABA. Shao and colleagues (Shao et al, 2020) specifically investigated GLP-1 and GABA pathways after discovering that a DPP-4 inhibitor sitagliptin in combination with GABA increased *β*-cell mass in the STZ-induced T1D mouse model (Liu et al, 2017). It was found that *β*-cell oxidative damage induced by high glucose and characterized by elevated expression of TxNIP can be attenuated by GABA, dependent on GLP-1R stimulation. A question was raised by the group on the role of the GABA and specifically GABA_B_ receptor in *β*-catenin-mediated signaling with additional stimulation of GLP-1R. An elegant study by Rachdi and colleagues in part addresses this question, as they find that the expression of functional GABA_B_ receptors in human islets is directly induced by GLP-1-cAMP signaling, which consequently initiates GABBR2 subunit mRNA expression (Rachdi et al, 2020). In clinical applications of combination therapies with DPP-4i, when the goal is to increase GLP-1R-mediated signaling, insufficient endogenous GLP-1 elevation could be an issue affecting treatment efficacy (Griffin et al, 2014).

#### 4 Gastrin-induced increase in β-cell mass and islet cell transdifferentiation

Increasing functional *β*-cell mass is one of the primary goals for intervention therapies in T1D. Proton-pump inhibitors (PPIs) are substituted benzimidazoles that inhibit acid secretion by blocking the hydrogen (H+) potassium (K+) adenosine triphosphate enzyme system (“proton pump”) in a variety of cells (Shin and Sachs, 2008), leading to a reduction in stomach acid production and an elevation in the blood levels of gastrin. Gastrin has pronounced effect on *β*-cell mass regeneration and reprogramming of pancreatic ductal cell pool and plays a substantial role in the process of differentiation of Neurogenin+ cells into endocrine cells during neonatal development (Gu et al, 2002; Collombat et al, 2006; Suissa et al, 2013). Possible mode of action of gastrin in the endocrine cells can be related to its function as both a conventional gut hormone and a powerful neurotransmitter in the gastrointestinal tract, central nervous system (CNS), and peripheral neurons. In human CNS and pancreas, gastrin primarily targets widely expressed G_i_-coupled CCKB receptor (Lee et al, 1993; Saillan-Barreau et al, 1999; Reubi et al, 2003) inducing antiapoptotic PKC-independent activation of mitogen-activated protein kinases (MAPK) and PI3K-AKT-mTOR signaling cascades (Kowalski-Chauvel et al, 1996; Ramamoorthy et al, 2004). During embryonic development, gastrin and CCKB receptors are highly expressed throughout the fundamental stage of endocrine cell differentiation in the developing pancreas, but their expression decreases after birth along with *β*-cell neogenesis (Bardram et al, 1990; Suissa et al, 2013; Téllez and Montanya, 2014). Gastrin expression is also peaked during the differentiation of human stem cells into insulin-secreting cells (Suissa et al, 2013), and gastrin-producing syngeneic mesenchymal stem cells was found to preserve *β*-cell mass in NOD mice (Gaudreau et al, 2022). These observations correspond with earlier studies that gastrin-induced signaling may induce *β*-cell regeneration in the NOD mouse model when coadministered with EGF (Suarez-Pinzon et al, 2005; Wang et al, 2012) and GLP-1RA (Fig. 3) (Suarez-Pinzon et al, 2008; Sasaki et al, 2015). Gastrin-related signaling pathways and biological activities in various cellular processes are reviewed in more detail elsewhere (Rehfeld, 2019; Zeng et al, 2020).

## IV Pharmaceutic interventions with individual targets in immune cells in type 1 diabetes

### A Pharmaceutically induced T-cell blockade leading to partial or full exhaustion

#### 1 Nonspecific immune therapy with ATG

**Fig. 4:**
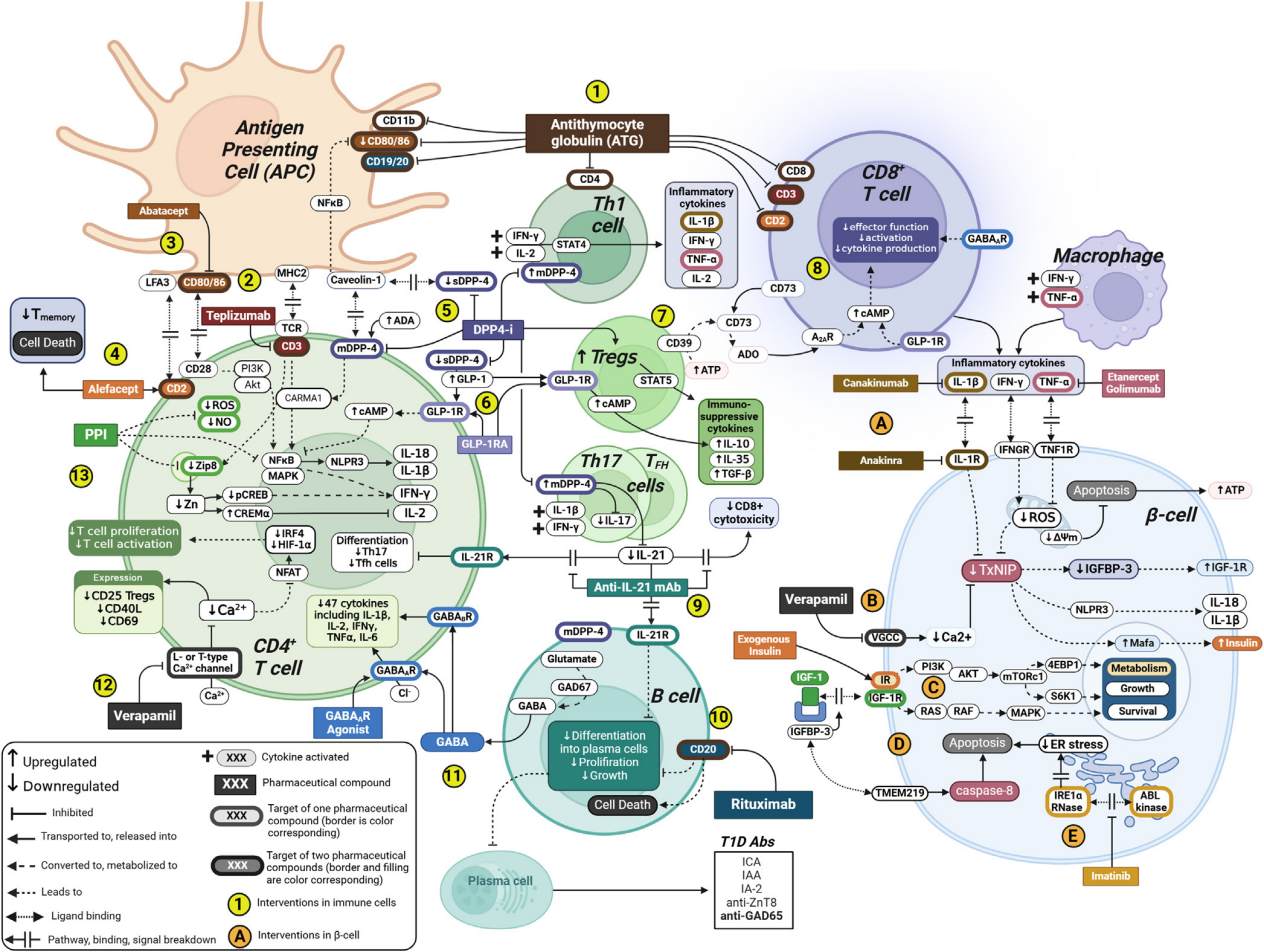
T1D targets for therapeutic interventions in immune cells. *Immune cells*: (1) Anti-thymocyte globulin (ATG) induces broad nonspecific immunosuppression that is primarily mediated through the recognition of a series of antigens expressed on human lymphohematopoietic cells, such as CD2, CD3, CD4, and CD8 expressed on T-cells, CD19 and CD20 expressed on B cells, and CD11b, CD80, and CD86 expressed on antigen-presenting cells (APCs). ATG achieves immunosuppression by eliminating lymphocytes in the recirculating pool through complement-mediated intravascular lysis, apoptosis, and antigen-dependent cell-mediated cytotoxicity. Low-dose ATG reduced HbA1c 2 years after therapy in recent-onset T1D patients. (2) Anti-CD3 monoclonal antibody (teplizumab) blockade of CD3 receptors in T-cells induces a state of anergy in certain T-cell populations, making them unresponsive to specific stimuli, and promoting regulatory T-cell functions. In recent-onset T1D patients, teplizumab was observed to preserve *β*-cell function and slow down C-peptide degradation. *Costimulatory signal blockade in T-cells:* (3) Abatacept is a CTLA-4/Fc fusion protein that prevents T-cell CD28 interaction with its CD80/86 ligand on APCs, thereby limiting immune system activation. Abatacept modified progression of T1D by significantly impacting CD4+ cell subsets, thereby delaying the decline of C-peptide and improving HbA1c in recent-onset T1D patients. (4) Alefacept is an anti-CD2 fusion protein that has a dual function, as it triggers PCD of activated memory T-cells and inhibits the interaction between leukocyte-function-associated antigen (LFA-3) and CD2, effectively preventing costimulatory signaling for the activation and proliferation of T-cells. In recent-onset T1D patients, 2 12-week courses of alefacept delayed C-peptide decline and depleted CD4+, CD8+ T-cells, and effector memory T-cells for over a year after cessation of therapy. (5) High expression of membrane-bound dipeptidyl peptidase-4 (mDPP-4) is associated with the differentiation of T lymphocytes into Th1 (IL-2, interferon gamma) and Th17 (IL-6, IL-17, and IL-22) cells and upon activation of B cells. DPP-4 inhibitors (DPP-4i) disrupt the mDPP4-caveolin-1 nuclear factor kappa B (NF*κ*B) activation pathway, which leads to a decrease in the expression of CD86 on antigen-presenting cells (APCs) and other monocytes. This limits the interaction between CD86 and CD28 on T-cells, resulting in a reduction in the proliferation and activation of antigen-specific T-cells. Additionally, DPP-4 inhibition prevents binding of mDPP-4 with adenosine deaminase (ADA) that would otherwise lead to the formation of a costimulatory CD3 signaling complex in CD4+ T-cells initiating CARMA-1 signaling. Furthermore, DPP-4 inhibitors prevent activation of Th1 cells, thereby leading to decreased secretion of proinflammatory cytokines: IL-1*β*, interferon gamma, TNF-*α*, and IL-2 from Th1 cells. *GLP-1:* (6) Activated T-cells express a higher number of functional glucagon-like peptide-1 receptors (GLP-1R) in human CD4+ T-cells and GLP-1R activation by GLP-1 receptor agonists (GLP-1RA) in Treg cells leads to increased IL-10 expression and enhanced cellular inhibitory function. *T regulatory cells (Treg cells):* (7) DPP-4 inhibition in CD4+ T-cells is found to promote the function of Treg cells and the production of the immunosuppressive cytokine TGF-*β*. Immunosuppressive functions of Treg cells are facilitated through CTLA-4 ligand binding to CD80/86 on APCs, expression of immunosuppressive cytokines: IL-10, TGF-beta and CD39-induced hydrolyzation of ATP to adenosine (ADO). Treg-derived ADO is a hydrolysis product of extracellular ATP cleaved in tandem by 2 Treg-associated ectonucleotidases, CD39 and CD73. (8) Most naïve CD8+ T-cells and a small number of mature CD4+ and Treg cells express CD73 on their surface. Enzyme-active CD73 is released from the CD8+ T-cell membrane upon activation, allowing Treg cell-driven ATP hydrolysis to occur, leading to adenosine formation. In immune cells, adenosine binds to the A2A receptor (A2aR), leading to the elevation of cAMP and interfering with the functions of activated T-cells and APCs, inhibiting their proliferation and cytokine production. *IL-21:* (9) IL-21 is produced primarily by CD4+ T-cells and is required for both Th17 cell differentiation and the generation of T follicular helper (Tfh) cells. IL-21 is the most prominent cytokine for the activation and differentiation of human B cells. IL-21 induces the differentiation of human naive and memory B cells into antibody-secreting plasma cells. Other cytokines, such as IL-4, greatly inhibit IL-21-driven plasma cell differentiation. IL-21 also directly regulates B-cell proliferation and apoptosis and can promote immunoglobulin production and isotype class switching. In addition, IL-21 signaling enhances the cytotoxicity of CD8+ T-cells and natural killer (NK) cells. A combination treatment of anti-IL-21 and GLP-1RA (liraglutide) preserved *β*-cell function in recent-onset T1D patients. This was demonstrated by a reduction in the concentration of C-peptide, as measured during a mixed-meal tolerance test (MMTT), from the baseline measurement to week 54 of treatment. *B cells*: (10) Anti-CD20 mAb (rituximab) effectively depletes mature B cells by various mechanisms inducing cell death, including DNA fragmentation, complement-dependent cytotoxicity (CDC), and programmed cell death (PCD). CD20 is reported to regulate B-cell differentiation and growth as well as adjusting Ca2+ transport. Notably, CD20 is detectable in pre-B cells to mature B cells but is absent in antigen-producing plasma cells. *GABA:* (11) Gamma-aminobutyric acid (GABA) has broad immune-modulating properties. It controls the release of cytokines from CD4+ T-cells and anti-CD3-stimulated peripheral blood mononuclear cells (PBMCs). Importantly, GABA suppresses the release of 47 cytokines in PBMCs from T1D patients and regulates pro- and anti-inflammatory cytokine production in a concentration-dependent manner. Engagement of the GABAA receptor (GABAAR) induces depolarization of the membrane potential, leading to inhibition of T-cell responses. B cells secrete GABA, which then inhibits inflammatory cytokine production in CD8+ T-cells and stimulates monocyte differentiation into IL-10-secreting immunosuppressive cells. *Calcium blockade*: (12) Lymphocyte calcium channel blockade may be an effective immunosuppressive strategy. Verapamil had a significant impact on T-cell activation by strongly inhibiting the expression of CD25 (which is typically present in Tregs), CD40L, and CD69. This inhibition is likely due to the failure of Ca2+-dependent transcription factors to activate gene transcription. Nuclear factor of activated T-cells (NFAT) is triggered by Ca2+, which also triggers the production of other transcription factors, including IRF4 and HIF-1*α*, that control the metabolic switch, cell cycle progression, and proliferation of activated human T-cells. Verapamil partially preserves *β*-cell function, as shown by C-peptide secretion in children and adolescents with recent-onset T1D. *Proton pump inhibitors*: (13) In immune cells, proton pump inhibitors (PPIs) suppress T-cell responses by decreasing the expression of the T-cell receptor (TCR)-activated membrane zinc transporter Zip8, thereby lowering the cytoplasm-free zinc (Zn) concentration. PPI-induced decrease in Zip8 expression increases transcription factor CREM*α*, which dramatically downregulates IL-2 production, while decreases in the transcription factor pCREB downregulates production of interferon gamma in lymphocytes. In monocytes, PPIs were found to reduce the production of several inflammatory cytokines: TNF-*α*, IL-1*β*, IL-6, and NF*κ*B. Moreover, PPIs inhibit the activation of neutrophils and monocytes and deplete intracellular and extracellular neutrophil reactive oxygen species (ROS) and nitric oxide (NO). PPI with DPP-4i in recent-onset T1D patients did not achieve C-peptide preservation, but due to high safety, PPI and DPP-4i have been suggested to be used in combination with other drugs. Preliminary results show that a combination of GABA, DPP-4i, and PPI as an adjunct to insulin therapy improves glycemic control in patients with T1D and elevates C-peptide levels in recent-onset T1D patients. *Beta-cells: Cytokine blockade.* (A) During the progression of T1D macrophages and T-cells invade the islets and secrete proinflammatory cytokines. The combination of TNF-*α* and interferon gamma synergistically induces *β*-cell apoptosis through activation of JNK/SAPK, resulting in the production of reactive oxidative species (ROS) and loss of mitochondrial transmembrane potential (ΔΨm). Proinflammatory cytokine blockade may act to prevent deleterious effects on *β*-cell survival and function in the islet microenvironment. Anti-inflammation and cytokine-modifying therapies showed varying degrees of effectiveness as TNF-*α* monoclonal antibodies (eg, Golimumab and Etanercept) postponed C-peptide loss in patients with recent-onset T1D. Canakinumab binds human IL-1*β* with high affinity and neutralizes its biological activity while Anakinra is an IL-1 receptor antagonist. Due to high safety, but insufficient efficacy in recent-onset T1D patients, canakinumab and anakinra have been suggested for IL-1*β* blockade as part of combination therapies. *Verapamil + IGF-1:* (B) Verapamil downregulates Ca2+ influx and thereby disrupts the formation of thioredoxin-interacting protein (TxNIP), reduces *β*-cell expression of IGF-binding protein 3 (IGFBP3) and thereby elevates IGF-1 induced signaling via increased IGF-1. (C) Stimulation of IGF-1R initiates PI3K/Akt signaling, which enables the activation of mTORC1. Upon activation mTORC1 phosphorylates the 4EBP1 protein, promoting cell growth, and the p70 ribosomal protein S6 kinase (S6K1), resulting in enhanced ribosomal biogenesis, mitochondrial biogenesis, and oxygen consumption. PI3K/Akt signaling also promotes *β*-cell-, but inhibits *α*-cell-related gene expression, as well as inhibiting *β*-cell apoptosis in the context of inflammatory cytokines and oxidative stress. Importantly, insulin receptor (IR) and IGF-1R are highly homologous and share PI3K/Akt and Ras/MAPK signaling pathways IR largely controls metabolism, whereas IGF-1R controls growth. (D) IGFBP-3 is a negative regulator of *β*-cell mass independent of IGF-1, which is a positive regulator. IGFBP-3 is a binding ligand to the death receptor TMEM219, which is widely expressed in islet *β*-cells. Bound TMEM219 triggers Caspase-8-mediated apoptosis of *β*-cells. (E) A 26-week course of a small tyrosine kinase inhibitor, imatinib mesylate (Gleevec, STI571) preserved *β*-cell function at 12 months in adults with recent-onset T1D. Imatinib acts as a *β*-cell protective drug, as it reduces ER stress and consequent *β*-cell apoptosis by inhibiting ABL kinase binding and hyperactivation of ER transmembrane kinase’s endoribonuclease (IRE1*α* RNase).

The polyclonal antithymocyte globulins (ATG) in the form of purified IgG fraction of sera from horses or rabbits immunized with thymocytes or T-cell lines have been used as potent and broad immune therapy for several decades (Mohty, 2007), primarily not only in organ transplantation but also in some autoimmune indications (Fig. 4). ATGs have a nonspecific and extensive antibody specificities ranging from direct immune response antigens to adhesion and cell trafficking ligands and finally heterogeneous pathways targets that can be found elsewhere (Gaber et al, 2010). Besides a well documented T-cell depletion caused by ATG directly targeting CD3, its nonexhaustive target list also includes HLA class I molecules expressed on all nucleated cells, CD2, CD4, CD5, CD6, CD8, CD25, and CD28 predominant in T-cells, CD80, CD86, and CTLA-4 in APCs, including several specific targets on activated B lymphocytes, such as CD5, CD20, CD28, and CD30. HLA class II molecule is, of course, a particularly interesting T1D target for ATG to explore in more detail, as its expression by *β*-cells as well as on APCs is hypothesized to initiate islet autoimmunity in disease pathophysiology. With such a broad spectrum of nonspecific immunosuppression, it is important to note that at low, submitogenic concentrations, therapy with ATG facilitates antibody-dependent cell cytotoxicity of preactivated T cells, but not resting cells.

Mechanistically, ATG-induced T-cell depletion is driven by antibody-dependent cytotoxicity and apoptotic Fas/Fas-L signaling pathway in mature lymphocytes (Genestier et al, 1998; Lagunas-Rangel, 2023). Earlier studies revealed that ATG activation-induced cell death (AICD) in mature-activated T cells could be initiated by continuous simultaneous signaling of monoclonal anti-CD3 antibodies and mitogenic lectins such as phytohemagglutinin (PHA; Kabelitz et al, 1993). Importantly, Fas-induced apoptosis required ATG preparation to include CD2 and CD3 antibodies for simultaneous stimulation, and the ATG-driven T-cell depletion was prevented by cyclosporin A, FK506, and rapamycin. Hypothetically, in a clinical setting, an adjustment in ATG dosing and composition can potentially be tailored to selectively target activated T cells and avoid substantial adverse events such as lymphocyte depletion and subsequent immunodeficiency. As such, low dosing of ATG and ATG in combination with pegylated granulocyte colony-stimulating factor (ATG + GCSF) in recent-onset T1D patients significantly reduced HbA1c. Additionally, ATG, but not ATG + GCSF, slowed the depletion of C-peptide at 1-year posttreatment (Haller et al, 2018). After 2 years of therapy, the levels of C-peptide were greater than placebo only in the ATG group, and both ATG and ATG + GCSF groups had significantly decreased levels of HbA1c (Haller et al, 2019). As a potential future treatment for T1D, it is important to note that low-dose ATG is not without significant limitations due to adverse events related to cytokine release or serum sickness. Although the side-effect profile of low-dose ATG is known and can generally be regulated, the intensity of serum sickness worsens with repeated use and may necessitate the use of follow-up treatments to maintain the outcome of the initial intervention. Development of innovative humanized ATG products holds promise to decrease occurrences of adverse events and improve clinical application as an intervention strategy.

#### 2 Specific immune therapy targeting CD3 with teplizumab

Teplizumab, an FcR-nonbinding anti-CD3 monoclonal antibody (anti-CD3), has been shown to induce alterations in the differentiation and activation state of CD3-expressing T-cells in the circulating pool, instead of depleting them. Teplizumab is one of the most widely investigated immune therapies in the field of T1D, with an overall of 14 ongoing, terminated, and finished studies accessible at https://clinicaltrials.gov/. In 2022, it was the first approved drug for the delay of T1D stage 3 onset in adults and children aged ≥8 years. A recent analysis by Herold and colleagues (Herold et al, 2023) showed significant preservation of C-peptide in response to teplizumab therapy based on pooled data from 5 clinical trials, a total of 609 patients (375 treatment/234 control) with stage 3 T1D. The same study conducted a safety analysis of 6 clinical trials with a total of 1018 patients (treatment 787/control 233) with stage 2 or 3 T1D. Almost all patients experienced grade 1 or 2 adverse events with the vast majority resolving without additional interventions; however, severe adverse events (grade 3) took place in 59% of treated patients compared with 22.9% of the control group. It is important to note that, as was the case with other immune therapies, although C-peptide depletion was significantly reduced after teplizumab intervention, the treatment was not able to increase or fully preserve it.

Notably, the complete mechanism of action of teplizumab has not yet been fully elucidated. Several research groups examined changes in T-cell subsets and phenotypes of 18 favorably responding stage 3 T1D from Antibody for Tolerance in Recently Diagnosed Type 1 Diabetes (AbATE) study with a total of 77 patients (52 teplizumab and 25 control) after 2 courses of teplizumab therapy, 1 year apart (Long et al, 2016, 2017; Tooley et al, 2016). Poststudy analyses discovered elevated CD8+ central memory T cells with particular gene signatures characterized by high levels of PD-1, TIGIT, KLRG1, and other eomesodermin (EOMES)-associated transcription factors correlated with successful response to teplizumab intervention. In the following sections, we will carefully consider that these anergic CD4+ and exhausted CD8+ T cells share phenotype similarities aligning with known mechanisms of tolerance and exhaustion in cancers (Schietinger and Greenberg, 2014).

More specifically, it was discovered that complex effects of anti-CD3 lead to transient rises in PD-1+Foxp3+ Treg cells, exhausted CD57-KLRG1+PD-1+, TIGIT+KLRG1+, EOMES CD8 T cells, and anergic CD57-KLRG1-PD-1+ CD4+ T cells. Rapid upregulation of programmed death protein (PD-1), also referred to as CD279, restricts both adaptive and innate immune responses, such as proliferation and survival in activated T cells (Keir et al, 2007) by blocking CD3/CD28-mediated PI3K/AKT (Parry et al, 2005), ZAP70/CD3*ζ* (Sheppard et al, 2004), and Ras/MEK/Erk signaling pathways (Patsoukis et al, 2012). Importantly, PD-1/PD-1L axis is crucially involved in tumor progression because cancer cells can exploit it to evade the immune system (Han et al, 2020). Blocking inhibitory signals that are important in cancer immunotherapy, but PD-1/PD-1 ligand pathway inhibition can also lead to T1D (Hughes et al, 2015; Roep et al, 2021). Given the important role of PD-1 overexpression to tumor-specific T-cell immunity, it might be plausible to follow-up on patients exhibiting an increase in PD-1 in response to anti-CD3 treatment, paying specific attention to documented cancer-associated PD-1 polymorphisms: PD-1.1 (rs36084323), PD-1.3 (rs11568821), PD-1.5 (rs2227981), PD-1.9 (rs2227982), and PD-1 (rs7421861; Salmaninejad et al, 2018).

Teplizumab-induced elevated expression of inhibitory immunoreceptor TIGIT is primarily associated with limiting antitumor responses in lymphocytes. It is expressed on APCs and competes with CD226 in NK and T cells, similar to how CTLA-4 prevents CD80 and CD86 receptors from interacting with CD28 and promotes direct T-cell inhibitory signaling cascades (Manieri et al, 2017). TIGIT expression and signaling in T cells have been shown to inhibit T-cell priming and reduce TCR-driven activation signals (Joller et al, 2011). Limited available research shows teplizumab-induced TIGIT expression in CD8+ T cells only. Lack of TIGIT in Tregs post-teplizumab treatment is an important immune target to consider for future intervention strategies because earlier research linked TIGIT expression in Tregs with direct inhibition of Th1 and Th17 responses (Johnston et al, 2014; Joller et al, 2014), reduction of interferon gamma (IFN-*γ*) in patients with multiple sclerosis, potentially by suppressing IL-12-induced PI3K-AKT-mTOR signaling cascade responsible for inducing dysfunctional proinflammatory Th1 Treg program (Ouyang et al, 2012; Lucca et al, 2019).

Finally, teplizumab-induced killer cell lectin-like receptor subfamily G member 1 (KLRG1) expression was found to be elevated in both CD8+ and CD4+ T cells. Consistent with CD57-KLRG1+PD-1+, TIGIT+KLRG1+ CD8 T cells described as displaying a partially exhausted phenotype (Long et al, 2016), Li and colleagues established that KLRG1+ T cells exhibit abundant proinflammatory cytokines and high level of Foxp3 expression but were found to contribute to the impaired antitumor immunity of memory T cells (Li et al, 2016). Additionally, in accordance with previous observations on the viral role in the manifestation of T1D and observations from the AbATE study on an increase in the detectable viral load in the treated group, Long and colleagues found that Epstein-Barr virus (EBV) reactivation significantly correlated with elevated transcription of EOMES genes in CD8+ T cells (Long et al, 2016). These findings demonstrate that pharmacologically induced alterations of phenotypes that lead to expansion of partially exhausted T cells, although known to be detrimental in tumor immunology and viral containment, might be considered advantageous reactions to immune therapy in T1D. An ongoing study on long-term safety for teplizumab (NCT04598893) is currently underway as an extension of the PRV-031-001 (PROTECT) phase 3 study to evaluate the efficacy and safety in stage 3 children and adolescents aged 8 through 17.

### B Pharmaceutically induced costimulatory signal blockade in T cells

#### 1 Abatacept-induced costimulatory blockade of CD28-CD80/86 signaling axis

Abatacept is a cytotoxic T-lymphocyte-associated protein Ig (CTLA4Ig) that prevents costimulatory signaling in T cells via CD80 and CD86 axis and substantially limits CD4+ and CD8+ T-cell activation. Mechanistically, abatacept inhibits T-cell activation via binding to CD80 and CD86 on APCs, thereby blocking the costimulatory interaction with CD28 on T cells. Another target is an inducible T-cell co-stimulator (ICOS) that becomes rapidly expressed on activated CD4+ and CD8+ T cells together with CD28 and CTLA-4, indicating its role in regulating the adaptive T-cell response (Watanabe et al, 2005). A 2-year clinical study on costimulation modulation in 112 patients (abatacept: 77 patients, placebo: 35 patients) with stage 3 recent-onset T1D showed a significant delay in the reduction of C-peptide area under the curve (AUC) (aggregate *P* = .0022) and HbA1c (aggregate *P* = .002; Orban et al, 2011). Insulin dosage decrease was significant at 6 and 12 months but leveled out to match the placebo at 24 months. In this trial, grade 3 and higher adverse events were recorded for 18 patients treated with abatacept, with the majority experiencing mild grade 1 or 2 adverse events, with an overall rate of all adverse events similar to that of placebo. A follow-up of these patients 1 year later showed that costimulatory blockade with abatacept decelerated the deterioration of *β*-cell activity and most notably sustained reduction in HbA1c levels (*P* < .005) 3 years after the diagnosis of T1D (Orban et al, 2014). In terms of prevention, an abatacept clinical study was conducted on 81 stage 1 T1D patients (abatacept: 35 patients, placebo: 46 patients), where the disease progression was not significantly delayed, but abatacept reduced PD-1+ T-follicular helper (Tfh) cells (*P* < .0001), increased naive CD4+ T cells, and also reduced the frequency of Tregs from the baseline (*P* = .0067), with complete return to baseline in 1 year after treatment. It is worth noting that the abatacept prevention trial had a primary endpoint effect on glucose tolerance instead of C-peptide AUC (Russell et al, 2023).

Clinical findings in abatacept prevention trial showed that abatacept primarily reduced the frequency of activated ICOS+ T follicular (Tfh) and T peripheral (Tph) helper CD4+ T cells, while maintaining the total number of CD4+ and CD8+ T cells in the naive state. Tfh CD4+ T cells, in particular, are crucial for B-cell responses, including the formation of memory B cells and the generation of antibodies and autoantibodies. Curiously, the investigators did not see a reduction in insulin autoantibodies and other autoantibodies after abatacept treatment. However, the decrease in ICOS+ CD4+ T cells indicated that costimulation blockage might have inhibited T-cell activation overall. Importantly, although abatacept effectively reduced the frequency of activated CD4+ T cells by blocking the CD28 axis, it did not induce either full or partial exhaustion of T cells. In both pediatric and adult patients, the frequency of these cells returned to baseline levels 12 months after treatment.

#### 2 Alefacept-induced costimulatory blockade of costimulatory CD2 signaling axis

Alefacept is a fusion protein composed of 2 LFA-3 molecules bound to IgG1 that binds CD2, expressed on CD4+ and CD8+ effector memory T cells, thereby interrupting CD2-mediated costimulatory activation. Inducing Remission in New-Onset T1D with alefacept (T1DAL; Rigby et al, 2013) study in stage 3 T1D patients (alefacept: 33 patients, placebo: 16 patients) showed a significant increase in the mean 4-hour C-peptide AUC (*P* = .019) and reduction in insulin demand (*P* = .02) after 12 months of treatment with no difference in HbA1c and mean 2-hour C-peptide AUC. All patients experienced at least 1 adverse event, mostly grade 1 or 2, some related to infection and/or major hypoglycemic events, 14 patients in alefacept group suffered from grade 3 and/or 4 adverse events compared with 9 taking placebo. Twenty-four months after 2 12-week treatments with alefacept, Rigby and colleagues subsequently reported on significant preservation of C-peptide AUC and reduction of exogenous insulin use in patients from the T1DAL study (Rigby et al, 2015).

Importantly, total white blood counts remained unchanged in both groups, but total lymphocytes and CD4+ and CD8+ T cells showed modest declines during the first and second course of treatment in the alefacept group, which rebounded by 78 weeks. The treatment significantly depleted CD4+ and CD8+ central memory and effector memory T cells, which were partially recovered at 24 months of follow-up. Importantly, alefacept therapy did not change the frequency of Tregs during and after the treatment. The 24-month findings of the T1DAL study support that a short-term focused immune intervention during the early stages may sustain pancreatic function in stage 3 recently diagnosed patients. Importantly, due to targeting particular T-cell memory subsets, alefacept treatment did not lead to cytokine release syndrome after administration and the reactivation of EBV, which was the case with some patients treated with anti-CD3 monoclonal antibodies (Keymeulen et al, 2010; Ambery et al, 2014).

### C Pharmaceutical inhibition of cytokines and corresponding receptors

Cytokines are promising prospective targets for immunotherapeutic interventions during different stages of T1D progression due to their direct implication in driving and regulating intricate multicellular connections across immune cells and pancreatic *β*-cells (Lu et al, 2020). Unfortunately, the precise functions of singular cytokines in the pathophysiology of T1D are still poorly understood, particularly in the context of many dysregulated cytokines actively contributing to inflammation and the advancement of the disease. Today, in the context of T1D, only a limited number of cytokines exhibit definitive and singular proinflammatory or anti-inflammatory properties exclusively. Such canonical cytokines that stimulate inflammatory processes have been identified in T1D sparking a significant interest in investigating whether inhibiting these cytokines might have therapeutic advantages. In the context of this review, we will consider pharmaceutical interventions related to canonical cytokines with proinflammatory functions, such as IL-1*β*, IL-21, and TNF-*α*.

IL-1*β* is a key mediator of inflammation and amplified adaptive immunity. It is secreted by several different cell types under metabolic damage and is recognized as a prominent marker and effector for different autoimmune conditions (Mohammadoo-Khorasani et al, 2016; Hu et al, 2020) including T1D. IL-1*β* role has been described as directly promoting the trafficking of immune cells into the islets and facilitating *β*-cell dysfunction and apoptosis (Mandrup-Poulsen et al, 2010; Sanda et al, 2010). IL-1*β*—induced inflammatory cell damage in T1D is prominently associated with NLRP3 inflammasome—caspase-1 activity, which detects cellular stress and cell membrane damage and is activated through Toll-like receptor mediated NF*κ*B pathway described in detail elsewhere (Grishman et al, 2012; Niț;ulescu et al, 2023). Islet *β*-cells are especially vulnerable to IL-1*β* induced apoptosis compared with other cell types due to their increased expression of IL-1 receptors (IL-1R; Böni-Schnetzler et al, 2009). Despite its established role in T1D pathogenesis, 2 clinical trials investigating IL-1*β* blockade in recent-onset T1D patients with canakinumab (treatment: 47 patients, placebo: 22 patients; adults and pediatrics) and anakinra (treatment: 35 patients, placebo: 34 patients; adults only) did not show efficacy as shown by 2-hour AUC C-peptide (Moran et al, 2013). Canakinumab was well tolerated with a high safety profile and did not result in elevated infections despite its potential anti-inflammatory effects. The anakinra trial was also considered well tolerated, but when compared with placebo, it had a significantly higher grade 2 adverse events rate, mostly related to dermatological conditions. Another open-label study on 14 insulin-resistant obese adults diagnosed as established T1D patients (no autoantibody or C-peptide data are available) treated with anakinra for 1 week showed a significant decrease in glucose levels (*P* < .01), improved insulin sensitivity weeks posttreatment (*P* = .02), and reduction of HbA1c (*P* < .01; van Asseldonk et al, 2015). Despite individual lack of efficacy, IL-1*β* blockers have been identified by animal model studies as prospective candidates for combination therapy with other compounds, such as anti-CD3 monoclonal antibody (mAbs) (Ablamunits et al, 2012) and GAD65-expressing plasmids (Pagni et al, 2014).

TNF-*α* is produced by many types of immune cells, including activated macrophages, CD4+ lymphocytes, natural killer cells, neutrophils, as well as neurons and pancreatic cells (Movahedi et al, 2004). Its significant elevation has been widely associated with several autoimmune conditions including T1D (Cavallo et al, 1991; Qiao et al, 2017). In animal models of T1D, TNF-*α* has been shown to directly potentiate toxic effects of other inflammatory cytokines such as IL-1*β* and interferon gamma and directly contribute to the destruction of *β*-cells (Mandrup-Poulsen et al, 1987; Eizirik, 1988; Pukel et al, 1988). Earlier clinical findings in 18 recent-onset T1D pediatric patients treated with a recombinant soluble TNF-*α* receptor fusion protein etanercept (9 patients) at 24 weeks showed a significant decrease in HbA1c levels (*P* < .05) and, notably, an increase in C-peptide AUC (*P* < .05; Mastrandrea et al, 2009). No severe adverse events were reported in either group, with cold symptoms twice as common in the treatment arm of the study. More recent study on Golimumab—a human IgG1-*κ* monoclonal antibody specific for human TNF-*α*, administered to 84 pediatric and adult patients with recent-onset T1D (treatment: 56 patients, placebo: 28 patients) at 52 weeks—showed significant preservation of 4-hour AUC C-peptide (*P* < .001) and lower insulin use (*P* = .001; Quattrin et al, 2020). Although the incidence of adverse events was recorded in 91% of the golimumab group compared with 82% of placebo, no serious events were recorded; the majority of cases were associated with infections in the treatment arm. Collectively, the findings from these studies provide evidence that TNF-*α* plays an important role in T1D and further support the idea of using TNF-*α* as a target for novel therapeutic approaches in careful conjunction with other pharmaceutical candidates.

### D Rituximab-induced blockade of CD20 in B-lymphocytes

CD20 is an activated-glycosylated phosphoprotein expressed on the surface of all B-lymphocytes (B-cells), but, notably, not plasma cells (Payandeh et al, 2019). CD20 is not associated with any physical binding ligands but is primarily responsible for B-cell proliferation, growth, and differentiation. Additionally, it is a part of the cell surface complex regulating the movement of calcium ions across the cell membrane (Hofmeister et al, 2000; Vacher et al, 2015; Berditchevski et al, 2021). These broadly spread molecular functions make CD20-targeted therapy a promising avenue in the treatment of cancer and autoimmune disorders. Rituximab is a CD20-specific chimeric monoclonal antibody (anti-CD20 mAb), which primarily functions by targeting and eliminating normal and malignant B-cells. It was initially approved for low-grade or follicular non-Hodgkin’s lymphoma and, more recently, for the treatment of rheumatoid arthritis (RA) unresponsive to anti-TNF-*α* therapies (Grillo-López et al, 1999; Edwards and Cambridge, 2006; Taylor and Lindorfer, 2007). The identified mechanisms of rituximab-induced cell death are broad and include the induction of antibody-dependent cell-mediated cytotoxicity (ADCC), complement-dependent cytotoxicity (CDC), and low levels of direct programmed cell death (PCD) by regulation of protein kinases, calcium influx, caspases, and BCL-2 family proteins (Bezombes et al, 2011).

A clinical trial in 87 recent-onset T1D adult and pediatric patients (treatment: 57 patients, placebo: 30 patients) showed that 4 rituximab infusions significantly preserved 2- and 4-hour AUC C-peptide and lowered insulin requirements (*P* < .001) and HbA1c levels (*P* < .001) at 1-year posttreatment (Pescovitz et al, 2009). Importantly, in the treatment group, CD19+ cells were depleted and gradually recovered by 12 months. The authors hypothesized that rituximab could have impaired B-cell antigen presentation necessary for T-cell activation and continued activity. No data on other immune cell subtypes is available, and the exact mechanism of action in T1D remains elusive. The treatment group was recorded to have more adverse events, majority grade 1 or 2, with grade 3 in 6 treated patients and 2 in placebo. Rituximab group at 1-year posttreatment displayed a significant drop in IgM levels (*P* < .001). Importantly, when compared with anti-CD3 mAb (teplizumab) clinical trial by Herold and colleagues (Herold et al, 2002), substantial distinctions are noted in the trial design. As such, in the Rituximab study, the patient cohort was older, and the treatment was started earlier (6 weeks vs 11 weeks postdiagnosis respectively), and the higher rate of adverse events with Rituximab was suggested to be related to the administration of infusions without continuous hospitalization.

### E Modifying immunity through autoantigens: GAD65 Alum

Substantial research is dedicated to the role of autoantigens for the elevation of immunologic tolerance as an alternative immune modifying therapy compared with immunosuppression in T1D, with primary antigen GAD65 being a predominant therapeutic target (Baekkeskov et al, 1982; Harrison, 2005). Alum-formulated human recombinant GAD65 (GAD65 Alum) was assessed in several clinical phase 2 and 3 studies alone and in combination with other pharmaceutical components with high safety profiles and no significant adverse events. However, achieving significant changes with GAD65 Alum in efficacy when it came to C-peptide preservation, clinical parameters, and protective effect of the pancreatic function persisted as a continuous challenge throughout these trials (Ludvigsson et al, 2008, 2012; Wherrett et al, 2011). Namely, there was a significant delay in C-peptide depletion after 30 months posttreatment in phase 2 study on 70 recent-onset patients between 10 and 18 years old (GAD65 Alum: 35 patients, placebo: 35 patients; Ludvigsson et al, 2008). However, in a TrialNet phase 2 study on 147 recent-onset patients aged 3–45 years old (3-dose regimen GAD65 Alum: 48 patients, 2-dose regimen GAD65 Alum, and 1-dose placebo: 49 patients, placebo: 48 patients) at 1-year posttreatment, no significance in 2-hour AUC C-peptide was observed (Wherrett et al, 2011). Similarly, no significance was achieved in stimulated C-peptide, HbA1c, insulin demand, and hypoglycemia rate was achieved in a phase 3 study on 327 recent-onset patients between 10 and 20 years of age (4-dose regimen GAD65 Alum: 111 patients, 2-dose regimen GAD65 Alum: 108 patients, placebo: 115 patients; Ludvigsson et al, 2012).

The administration route of the GAD65 Alum intervention warrants further examination, given the disparate effects observed in early clinical trials that utilized 100 *μ*g GAD subcutaneously, in contrast to more recent studies employing 4 *μ*g intralymph node injections (Hannelius et al, 2020; Ludvigsson et al, 2021b; Dietrich et al, 2022; Nowak et al., 2022a, Nowak et al., 2022b). Clinical data indicate that GAD65 Alum given intralymphatically resulted in higher GAD65 autoantibody (GADA) levels compared with larger doses supplied subcutaneously. Various routes of delivery were often combined with an alternate secondary treatment (eg, vitamin D, ibuprofen, and etanercept discussed in sections Combining vitamin D with GAD65 Alum andibuprofen and Combining vitamin D with GAD65 Alum and etanercept) to provide a more conducive environment for eliciting Th2 and Tr1 regulatory responses. Mechanistic assessment of the treatment effect in immune cells is somewhat unexpected, as clinical studies note that GAD65 Alum administration results in a rise of GADA levels with subsequent decline at 9 months posttreatment (Ludvigsson et al, 2008, 2012; Ludvigsson et al, 2021a). Of note is also upregulated inflammatory cytokine secretion in response to GAD65 Alum treatment, particularly of IL-1, IL-17, interferon gamma, and TNF-*α*. This would result in a cytokine-driven autoimmune assault on the *β*-cells (Ludvigsson et al, 2008, 2020). Interestingly, despite inconsistent findings from clinical trials, Beam and colleagues conducted a comprehensive Bayesian meta-analysis of individual patient data from these 3 studies and came to a conclusion that 2-dose of GAD65 Alum has a probable 15%–20% reduction in the C-peptide decline at 1-year posttreatment (Beam et al, 2017).

### F Modifying immunity with vitamin D (vitamin D)

Vitamin D is a secosteroid, which, in its biologically active state, 1,25-dihydroxyvitamin D3 [1,25(OH)2D3] (vitamin D3), functions as an endocrine and immunoregulatory hormone. Its major role is to regulate calcium and phosphate levels in the serum to facilitate the maintenance of bone and mineral homeostasis. Vitamin D can be synthesized by immune cells and directly control the proliferation and specialization of several kinds of cells. As such, it plays a significant role in paracrine and autocrine signaling regulating innate immune response through vitamin D receptor (VDR) expressed on macrophages, APCs, and T and B cells (Adorini and Penna, 2008; Hewison, 2010; Cutolo et al, 2023). In particular, vitamin D has a direct effect on CD4+ T-cell polarization through promoting protective Th2 phenotype development (Boonstra et al, 2001). A number of studies outlined various possible roles and benefits of vitamin D and its supplementation in T1D and other autoimmune conditions (Kivity et al, 2011; Hou et al, 2021; Cutolo et al, 2023). Numerous studies reported vitamin D positive effects as immunomodulator, such as reduction in MHC class I expression on *β*-cells and its protective effects against cytokine-induced apoptosis in the pancreatic islets (Hahn et al, 1997; Riachy et al, 2002, 2006). A comprehensive review of vitamin D as an intervention in T1D is discussed in detail elsewhere (Yu et al, 2022). Clinical findings from 13 clinical trials show that intervention treatment with vitamin D in different active forms shows a trend for C-peptide preservation but is not sufficient to significantly alter the course of the disease (Yu et al, 2022). Nevertheless, a number of beneficial effects have been reported by different studies, including a reduction in insulin demand at 3 months (Pitocco et al, 2006), transient preservation of C-peptide (Gabbay et al, 2012; Sharma et al, 2017), reduced PBMC reactivity to GAD65 and proinsulin (Federico et al, 2014), lower TNF-*α* levels at 12 months (Nwosu et al, 2022), and improved glycemic control (Panjiyar et al, 2018). A clinical study on cholecalciferol adjunct to insulin in 38 recent-onset T1D aged 7–30 years old (treatment:19 patients, placebo: 19 patients) had no adverse events and yielded transient preservation of stimulated C-peptide at 12 months (*P* = .01) with a reduced rate of degradation at 18 months (Gabbay et al, 2012). Importantly, when assessing specific immune targets, authors point out cholecalciferol-induced downregulation of inflammatory chemokine IFN-*γ*-inducible protein 10 kDa (CXCL10), which was significantly higher at baseline (*P* = .02), and increased expression of chemokine ligand 2 (CCL2) at 12 months (*P* = .04), both factors connected to significant treatment-induced elevation in Treg numbers at 12 months of follow-up (*P* = .04). From an immunomodulatory perspective, there are important effects of vitamin D treatment to consider, including low toxicity and high safety profile, which make it a compelling candidate for a variety of combination treatments.

### G Combining vitamin D with GAD65 Alum and ibuprofen

Two-dose regimen of GAD65 Alum, vitamin D, and ibuprofen was assessed on 64 patients with recent-onset T1D aged 10–18 years old (full treatment: 16 patients, placebo + GAD65 Alum + vitamin D: 16 patients, placebo + GAD65 Alum double dose + vitamin D: 16 patients, placebo: 16 patients; Ludvigsson et al, 2020). No significant changes were observed in 1.5- and 2-hour AUC C-peptide, HbA1c, and insulin demand, but important distinctions were made on best responding patients’ baseline characteristics such as GADA levels, insulin dose/kg per 24 hours, and HbA1c.

### H Combining vitamin D with GAD65 Alum and etanercept

As seen from an earlier review of recombinant soluble TNF-*α* receptor fusion protein-related interventions, GAD65 Alum has been administered in combination with etanercept and vitamin D in an open-label pilot study on 20 patients (Ludvigsson et al, 2021a). Vitamin D was also tried in a dual combination with GAD65 Alum in a phase 2b study in 109 recent-onset T1D patients aged 12–24 years old (treatment: 57 patients, placebo: 52 patients; Ludvigsson et al, 2021b). Although the primary endpoint of 2-hour AUC C-peptide was not met, this particular study makes an important patient subgroup differentiation based on HLA haplotypes, thereby promoting a precision disease-modifying approach. As such, a specific subgroup of 29 HLA-DR3-DQ2 patients in treatment significantly preserved C-peptide AUC against 17 patients with the same haplotype in placebo (*P* = .0078).

Unexpectedly, the combination of GAD65 Alum with TNF-*α* blocker etanercept led to transient IL-4 elevation, continuous upregulation of IL-17 (*P* = .004), and a significant increase in IFN-*γ* and TNF-*α* (*P* = .002 and *P* = .02, respectively) at 1 month posttreatment, failing to produce an inhibitory immunomodulatory effect in patients with recent-onset T1D. Mass cytometry profile of PBMCs from 12 recent-onset T1D patients 15 months post-GAD65 Alum administration into an inguinal lymph node showed, among other changes, a significant increase in Tfh cells, which are heavily engaged in IL-17 and IL-21 secretion, upregulation of PD-1+CD8+ T-cells, and reduction in naïve memory B-cells (Barcenilla et al, 2021). Twelve recent-onset T1D patients administration of GAD65 Alum as a preventative intervention in stage 1 healthy pediatric patients with antibody positivity (treatment: 13 patients, placebo: 13 patients) yielded promising preliminary results and showed significantly lower levels of T cells (*P* = .008), T-helper cells (*P* = .014), and cytotoxic T-cells (*P* = .023) 26 months posttreatment (Salami et al, 2022).

## V Pharmaceutic interventions with individual targets in islet endocrine cells in type 1 diabetes

### A Targeting β-cell homeostasis through G protein-coupled receptors

G protein-coupled receptor (GPCR)-mediated signaling is responsible for crucial cellular regulation processes, including cAMP production, PI3K, and Ras activation to name a few (Janetopoulos et al, 2001; Lagerström and Schiöth, 2008; Hauser et al, 2017; Lagunas-Rangel, 2022). The major downstream signaling cascades, G_S_-GPCR-cAMP and G_i/o_-GPCR-PI3K-AKT, are activated by a number of pharmaceutical compounds capable of binding these ligands and regulating major cell functions responsible for viability in T1D (Fig. 3).

#### 1 GLP-1R-mediated short-term rescue of GSIS by elevating G_s_-GPCR-cAMP-PKA/Epac2- and PDX1-associated cell function

GSIS is one of the fundamental regulatory mechanisms of insulin exocytosis in the *β*-cell that presents a key pharmaceutical target in diabetes. As discussed earlier, incretins initiate G_s_-GPCR-cAMP signaling by phosphorylation by cAMP-dependent protein kinase A (PKA), and the cAMP-binding protein Epac2 (Seino et al, 2009). Two widely available antidiabetic therapeutic agents, GLP-1R agonists and DPP-4 inhibitors, have been developed with a particular focus on cAMP-induced signaling in *β*-cells. Mechanistic targets of these drugs can be found on Fig. 3. Studies on GLP-1-cAMP-PKA-induced signaling show that GLP-1RA can activate and upregulate the expression of pancreatic duodenal homeobox-1 protein (PDX1) responsible for *β*-cell phenotype and function (Wang et al, 2001). When used individually, the GLP-1RA efficacy in T1D adults has been assessed in 11 clinical trials with moderate beneficial effects on reduction in HbA1c levels and insulin demands (Tan et al, 2023). Importantly, GLP-1RA did not increase the incidence of serious adverse events, such as severe hypoglycemia or diabetic ketoacidosis. Together, these findings coupled with an understanding of pathways to be targeted by GLP-1RA make these drugs appealing candidates for a combination treatment of T1D.

#### 2 Targeting G_i/o_-GPCR signaling to amplify PI3K-AKT survival pathways

Today, there are several pharmacologically addressable G_i/o_-GPCRs that are expressed on *β*-cells, such as somatostatin, gastrin/cholecystokinin (CCKB) receptors, *α*2A-adrenergic receptors (ADRs), D2-like dopamine receptors (DRDs), and GABA_B_ receptors. G_i/o_-GPCR-initiated signaling inhibits cAMP transmission but is responsible for a significantly higher generation of PI3K, a lipid kinase that spreads intracellular signal cascades and regulates a variety of cellular processes. PI3K recruits protein kinase B (AKT), a downstream effector of multiple growth, proliferation, survival, metabolism, and autophagy pathways, such as mTORC1 (Jean and Kiger, 2014). A recent study by Dickerson and colleagues illuminates a primary mechanism of how G_i/o_-GPCRs control *β*-cell electrical excitability by inducing membrane hyperpolarization, thereby directly affecting intracellular calcium handling and insulin secretion (Dickerson et al, 2022). The mechanism describes the regulation of electrogenic NKAs by GPCR-induced signaling, whereby G_s_-GPCR-cAMP-PKA activation effectively inhibits NKA function, leading to membrane depolarization and calcium influx. Stimulation of G_i/o_-GPCRs and tyrosine kinases (STKs, insulin receptors) on the contrary, hyperpolarizes cell membrane via activation of NKAs and initiates closure of voltage-gated calcium channels (depicted in Figs. 1 and 3). Understanding these processes is particularly important because it will allow a tailored intervention targeted specifically at 2 counter-regulatory mechanisms that regulate *β*-cell function, electrical excitability, and calcium handling by modulating NKA activity.

#### 3 Competitively inhibiting glucagon receptor signaling

On another end of the pharmaceutical intervention spectrum are GPCR antagonists. Glucagon receptor is G_s_-GPCR and acts through cAMP-PKA signaling as a part of paracrine crosstalk in the islets. Earlier works identified glucagon receptor antagonism as a promising pathway for overcoming complete insulin deficiency and maintaining appropriate glycemic control in animal models (Gelling et al, 2003; Wang et al, 2015b). Human immunoglobulin G2 (IgG2) mAb volagidemab competitively blocks glucagon receptors and inhibits its downstream signaling (Pettus et al, 2022). volagidemab 12-week treatment in 18–65 years T1D patients (volagidemab 35 mg: 2 patients, volagidemab 70 mg: 2 patients, placebo: 27 patients) failed to reduce the daily insulin use but showed a significant reduction in HbA1c and tolerable safety profile. Importantly, all participants had a fasting C-peptide of <0.7 ng/mL, and although authors note no increase in hypoglycemia in treated patients, significant increases in serum transaminases, LDL cholesterol, and blood pressure were recorded (Pettus et al, 2022).

#### 4 Inhibiting G_s_-GPCR to amplify AMPK survival pathway

Blunting glucagon downstream signaling by means of reducing cAMP accumulation and activation of AMP-activated protein kinase (AMPK) is a known mechanism of another antidiabetic drug: metformin (Miller et al, 2013; Cao et al, 2014). The detailed glucoregulatory mechanisms and modes of action of metformin are extensively discussed elsewhere (Foretz et al, 2019, 2023). Under cellular energy deficit, AMPK deactivates biosynthetic pathways and other nonessential processes that consume ATP and activates alternative catabolic pathways that generate ATP to restore energy balance (Hardie, 2014). Suppression of mitochondrial complex 1 results in a reduction in ATP levels and an elevation in the AMP/ATP ratio, which subsequently directly inhibits gluconeogenesis (Fig. 3) (Foretz et al, 2010).

Previous studies have demonstrated that metformin exerts its effects through mechanisms that are both AMPK-dependent and AMPK-independent by impeding mitochondrial respiration, typically activated by G_s_-GPCR-cAMP downstream signaling (Owen et al, 2000), and inhibiting lysosome-related mechanisms (Rena et al, 2017). Independently of AMPK signaling, metformin acts as a potent mTORC1 inhibitor (Kalender et al, 2010), conceivably by means of inhibition of V-ATPase-Ragulator complex located in lysosomes and endosomes (Ma et al, 2022). It is an intriguing therapeutic target to consider for managing cellular metabolism and energy stress in the islets as is required for LKB1-mediated AMPK and mTORC1 activation, thereby providing a switch between catabolism and anabolism in the cell (Zhang et al, 2014). Although mechanistic targets of metformin in pancreatic islets still have not been completely elucidated, some studies have shown their protective and proliferative functions in *β*-cells in vitro (Marchetti et al, 2004; Tajima et al, 2017).

### B Imatinib-induced mediation of β-cell ER stress

A first-in-class tyrosine kinase inhibitor successfully applied in chronic myeloid leukemia—imatinib—showed promise as a therapeutic agent for T1D, specifically addressing ER stress and glucose homeostasis in *β*-cells in preclinical studies (Hägerkvist et al, 2007; Han et al, 2009; Morita et al, 2017; Attwood et al, 2021). Imatinib primarily is known for inhibiting abelson murine leukemia viral oncogene homolog 1 (ABL1) kinase but is also reported to inhibit other types of tyrosine kinases that play a role in various autoimmune indications (D’Aura Swanson et al, 2009; Azizi and Mirshafiey, 2013). In *β*-cells, during prolonged ER stress, cytosolic ABL kinase colocalizes to the ER membrane and hyperactivates ER transmembrane kinase endoribonuclease’s (IRE1*α*) enzymatic activities to critical levels, potentiating the proapoptotic terminal-UPR, mRNA splicing, ER-localized mRNA decay, induction of TxNIP, and apoptosis (Fig. 4). Imatinib disrupts ABL kinase transport to the ER and its subsequent stimulatory interaction with IRE1*α*, thereby preventing proapoptotic downstream signaling in the ER and promoting *β*-cell survival in T1D (Morita et al, 2017). In phase 2 clinical trial, 26-week imatinib therapy administered to 18–45 years old recent-onset T1D patients (treatment: 43 patients, placebo: 21 patients) showed significant preservation of C-peptide at 12 months when compared with placebo, but not at 24 months (Gitelman et al, 2021). Notably, 71% of the treatment group experienced adverse events as a result of the treatment, with 13% discontinuing therapy. However, the efficacy in diagnosed T1D patients clearly demonstrates that imatinib was able to pharmacologically target at least 1 crucial metabolic pathway in the *β*-cells during the onset of T1D, which may significantly contribute to developing a combination approach to alter the progression of the disease.

## VI Individual pharmaceutical compounds simultaneously targeting *β*-cells and immune cells

### A Verapamil-induced activating voltage-gated calcium inhibition

#### 1 Calcium-induced inhibition of TxNIP assembly in β-cells

Xu and colleagues show that 1 way to significantly decrease proapoptotic TxNIP expression in *β*-cells under glucotoxicity can be achieved by using first-generation calcium channel blockers, such as verapamil and diltiazem (Xu et al, 2012). The authors indicate that downregulation of TxNIP expression is primarily mediated by overall downregulation of cytoplasmic calcium levels, nonspecific to class or type of a particular calcium channel blocker used (Fig. 4). Verapamil, specifically, was found to prevent nuclear transcription of TxNIP in the *β*-cell by blocking off E-box motif that serves as a binding site for ChREBP, significantly reducing its nuclear presence. Importantly, verapamil was shown to selectively reduce TxNIP expression only under hyperglycemic conditions, making it a promising intervention in patients suffering from diabetes.

Importantly, these findings were later successfully translated into clinical studies with verapamil showing improvement in *β*-cell function of recent-onset pediatric (Forlenza et al, 2023) and adult patients (Ovalle et al, 2018). In a more recent study, Xu and colleagues also discovered that verapamil-induced downregulation of TxNIP promotes the elevation of insulin-growth factor 1 (IGF-1) and a significant decrease in the expression of IGF-binding protein 3 (IGFBP3; Xu et al, 2023). The authors note the particular significance of the verapamil-enhanced IGF-1 signaling pathway in restoring appropriate *β*-cell function. This is an important observation, because although insulin receptor (IR) and IGF-1 receptor are highly homologous and share PI3K-AKT and RAS-MAPK signaling pathways, each receptor is responsible for distinct activities within the cell (Cai et al, 2017). IR is responsible for cell metabolism, whereas IGF-1-induced signaling controls mTORC1 phosphorylation of 4EBP1 protein responsible for growth and proliferation and p70 ribosomal protein S6 kinase (S6K1), mitigating oxidative stress, ribosomal, and mitochondrial biogenesis (Proud, 2007). IGFBP3 was recently found to be elevated in diagnosed recent-onset and established T1D patients and its direct binding of TMEM219 receptor initiates caspase-8 induced *β*-cell dysfunction and apoptosis (D’Addio et al, 2022). Therefore, a calcium blockade by pharmaceutical intervention can effectively target not 1, but several *β*-cell fragilities associated with hyperglycemia-induced metabolic stress through the downregulation of proapoptotic factor TxNIP and reduce extracellular proapoptotic signaling via IGFBP3. Considering these particular signaling mechanisms can also be beneficial when identifying combinational treatment synergies for other pharmaceutical agents that are also able to downregulate TxNIP expression and elevation of IGF-1, such as GLP-1RA (Xie et al, 2014).

#### 2 Calcium blockade in immune cells

Calcium transport holds a central role in immunomodulation of T-cell function and adaptive immunity as a whole. The beneficial effects of L-type calcium channel blocker verapamil in T1D clinical trials and its effect on endocrine cells are described earlier in the paper. The outstanding question to consider is whether it might also have a role to play in immunomodulation through targeting Ca2+ transmission in immune cells. Importantly, the primary source of Ca2+ in T-cells is the Ca2+ release-activated Ca2+ channel (CRAC), which includes ORAI1 and ORAI2 proteins (Prakriya et al, 2006; Vaeth et al, 2017b). Although ORAI channels are resistant to verapamil, secondary Ca2+ sources, including T and L-type VGCC, have also been proposed to contribute to calcium-driven signaling cascades in T-cells (Fenninger and Jefferies, 2019). Altogether, these calcium fluxes are essential for the recruitment and accumulation of the nuclear factor of activated T-cells (NFAT) isoforms, as well as other transcription factors regulating clonal growth, cell survival, differentiation, and cytokine synthesis (Feske, 2007; Vaeth et al, 2017a).

Veytia-Bucheli and colleagues provide a comprehensive in vitro study on its role in T-cells with several interesting discoveries that can significantly contribute to increasing efficacy in future clinical studies (Veytia-Bucheli et al, 2022). In particular, the authors found that dose-dependent administration of verapamil in response to CD3 stimulation alone exerts complete inhibition of T-cell proliferation, and potently inhibits the expression of CD25, CD40L, and CD69 surface ligands. It was also found to impair the polarization of Th1, Th17, and Th2 along with the secretion of associated cytokines (eg, interferon gamma, TNF-*α*, IL-2, IL-17, IL-4, and IL-10). Most notably, this inhibitory effect is significantly negated when the cells are stimulated with both CD3 and CD28. These findings demonstrate that costimulatory signaling in T cells activates supplementary pathways that circumvent TCR-activated calcium-dependent processes that are restricted by verapamil, thereby partially recovering T-cell activity. Building on the existing promising clinical findings of verapamil intervention in recent-onset T1D patients (Ovalle et al, 2018; Forlenza et al, 2023), these in vitro discoveries provide opportunities for more focused pharmaceutical intervention strategies, such as including costimulatory signal blockers (eg, abatacept) in combination therapies that include verapamil as one of the components.

### B Baricitinib-induced inhibition of JAK-STAT-IRF-1 pathway

#### 1 Mediating cytokine-induced damage in β-cells

Some groups have identified a detailed activation pathway of the ER stress sensors in the *β*-cells, particularly in response to inflammatory cytokines (Gysemans et al, 2008; Moore et al, 2011). IFN-*γ* triggers downstream signaling, typically via Janus kinase (JAK) that directly induces apoptosis via the activation of the signal transducer and activator of transcription (STAT) and binding to IFN-*γ* regulatory factor-1 (IRF-1) promoter. Importantly, IFN-*γ* upregulates the expression of MHC class I expression on the surface of *β*-cells (Baldeón et al, 1997); however, direct blockade of IFN-*γ* receptors on the *β*-cells did not impact diabetes progression (Thomas et al, 1998). This may be indicative of other interferons and *γ*-chain cytokines signaling through the JAK-STAT axis in *β*-cells and its importance in the pathogenesis of autoimmune diabetes. Mechanistically, inhibitors of JAK1/JAK2 inhibited JAK-STAT pathway in mouse and human *β*-cells and successfully blocked the effect of cytokines by downregulating MHC class I and reversing diabetes in NOD mice (Trivedi et al, 2017; Ge et al, 2020). Notably, these results successfully translated into a phase 2 clinical study, as daily treatment with a single tablet of baricitinib for over 48 weeks successfully preserved C-peptide in recent-onset T1D patients and reduced exogenous basal and prandial insulin intake with minimal adverse events (Waibel et al, 2023). The subsequent sections of this review article covering JAK-STAT-related autoimmune interventions in T1D provide a more comprehensive analysis of findings in Waibel and colleagues’ study. At present, 4 JAK inhibitors, namely baricitinib, tofacitinib, upadacitinib, and ruxolitinib, received approval for clinical use in the treatment of other indications, namely RA and myelofibrosis.

#### 2 Blocking interferon gamma and γ chain cytokines signaling in immune cells

JAK inhibitors exert large potential as a comprehensive intervention for T1D, as they are uniquely positioned to address immune as well as endocrine dysfunctional JAK-STAT molecular pathways involved in disease pathophysiology. This is reflected in a recently published baricitinib phase 2 clinical study on 91 recent-onset T1D patients aged between 10 and 30 years old (treatment: 60 patients, placebo: 31 patients; Waibel et al, 2023). Most notably, at the 48-week assessment, the mixed-meal-stimulated C-peptide in baricitinib group was significantly higher than placebo (*P* = .001) and, importantly, higher than at baseline. Secondary endpoints also showed lower levels of HbA1c compared with placebo and nonsignificant reduction in insulin demand compared with baseline and placebo. Authors hypothesize that the treatment efficacy may be higher in patients with more residual C-peptide function, meaning initiating treatment immediately after diagnosis or preventatively in asymptomatic stage 2 patients. Interesting observations were made on mechanistic effects of baricitinib in immune cells. Waibel and colleagues recorded a significant reduction in the number of CD8+ T-cells and IL-21–stimulated phosphorylated STAT3. These observations are consistent with previous studies of JAK inhibitors in animal models (Trivedi et al, 2017; Ge et al, 2020). JAK inhibitors were seen to block JAK-STAT downstream signaling cascades initiated by T1D-associated IFN-*γ* and *γ* chain cytokines, including IL-21 (Sutherland et al, 2013; Ge et al, 2020).

Importantly, baricitinib is an orally administered tablet with an acceptable safety profile, which is additionally confirmed by a consensus statement for the treatment of immune-mediated inflammatory diseases with JAK inhibitors (Nash et al, 2021). The authors attribute low drug toxicity to partial inhibition of JAK at the highest doses of baricitinib and no effect on JAK at lower levels. These data combined suggest that JAK inhibitors, including baricitinib, have broad target ranges through diverse JAK-STAT roles regulating inflammation in immune and *β*-cells. As a result, these inhibitors appear to be more successful in combating the development of T1D compared with selective pharmaceutical ligands or cytokine blockers.

### C DPP-4 inhibitors

#### 1 Preventing degradation of incretins and associated G_S_-GPCR signaling in β-cells

As mentioned earlier, DPP-4i are widely used antidiabetic agents primarily administered to T2D patients to prevent the degradation of incretins, such as GIP and GLP-1, by DPP-4 enzymes. The importance of incretin-stimulated G_s_-GPRC signaling in *β*-cells is described earlier in this review (see section Incretin rescue of GSIS). A meta-analysis on DPP-4i used as intervention therapy in 253 diagnosed T1D patients pooled from 5 randomized controlled trials showed a trend to decrease in HbA1c levels and improvement in insulin demands and glycemic control (Wang et al, 2018). Despite inconclusive clinical benefits when used as an individual component, DPP-4i are associated with a low incidence of minor adverse events and a high safety profile in adults, elderly, and pediatric patients (Gadsby, 2009; Jalaludin et al, 2022; Shankar et al, 2022; Nagao et al, 2023). In T2D clinical trials, sitagliptin specifically has been consistently observed to lower HbA1c and retain a high safety profile not only as a monotherapy, but in combination with metformin, pioglitazone, sulfonylurea, and insulin (Charbonnel et al, 2006; Raz et al, 2006; Rosenstock et al, 2006; Vilsbøll et al, 2010; Moses et al, 2016).

#### 2 Pharmaceutically inhibiting the costimulatory CD26/mDPP-4 signaling axis in CD4+ T cells

CD26 is a T-cell costimulatory activation antigen with membrane-bound dipeptidyl peptidase-4 (mDPP-4) involved in various cellular processes through binding to several ligands, including adenosine deaminase (ADA), CD45, fibronectin, plasminogen, and caveolin-1 (Huang et al, 2022). CD26 is not only limited to T-cell functions but is also involved in B-cell activation (Bühling et al, 1995). Increasing evidence indicates that DPP-4 activity in the serum and mDPP-4 expression in immune cells play a role in the pathophysiology of autoimmune diseases (Yoon et al, 2021). As with other costimulatory pathways, such as CD28, costimulation of mDPP-4 in combination with an antigen-specific stimulus provided by a TCR-CD3 complex leads to maximal T-cell activation (Tanaka et al, 1994; ElTanbouly and Noelle, 2021).

Although data on DPP-4i impact in immune cells of T1D patients remain limited, Varga and colleagues discovered that in T1D patients, serum DPP-4 enzymatic activity is elevated, but mDPP-4 expression on lymphocyte membranes is reduced, which may indicate a novel T effector cell regulatory malfunction (Varga et al, 2011). Notably, serum DPP-4 levels in T1D patients were also found to correlate with diabetes duration (Wang and Hiesinger, 2013; Hirao et al, 2020). A number of recent studies show the involvement of CD26-mediated signaling in the differentiation of CD4+ T-cells into Th1 Th2 and Th17 subtypes, thereby promoting the secretion of IL-2, interferon gamma, IL-6, IL-17, and IL-22 inflammatory cytokines (Pinheiro et al, 2017; Zhao et al, 2021). Meta-analysis of 8 controlled T2D clinical trials showed that DPP-4i (sitagliptin and vildagliptin) are associated with significant reductions in TNF-*α*, an important mediator for T1D-related inflammatory processes (Atkin et al, 2017). Consistent with these findings and earlier studies on DPP-4i-induced alteration of the T-cell phenotype in T2D patients (Aso et al, 2015), Wang and colleagues show that inhibition of CD26 with sitagliptin in patients with LADA (20 sitagliptin, 20 control) significantly upregulates the ratio of Tregs to CD4+ T cells (*P* = .016) and downregulates the percentage of Th2 at 6 months (*P* = .015), 12 months (*P* = .012) and Th17 at 12 months (*P* = .020) of treatment (Wang et al, 2019).

#### 3 Immune cell processes triggered by mDPP-4 activation

Regarding T1D-driven autoimmunity, mDPP-4 expression is highest in activated Th17 and Th1 and to a lesser extent in Th2 CD4+ T-cell subsets, presenting particular targets for pharmaceutical intervention (Willheim et al, 1997; Lun et al, 2007; Bengsch et al, 2012). In CD4+ T cells, ligation of mDPP-4 and CD45 complex provides a costimulatory signal for T-cell activation that enhances the downstream phosphorylation of CD3-ζ, p56lck (LCK), and ZAP-70 (Ishii et al, 2001; Katz et al, 2017).

Additionally, mDPP-4 forms complexes with ADA, thereby significantly depleting the local concentration of adenosine that plays an important role in inhibiting T-cell proliferation and IL-2 secretion as an independent immunoregulatory mechanism (Dong et al, 1996, 1997). Selective mechanical studies in immune cells identify caveolin-1 as a functional receptor for mDPP-4 (Ohnuma et al, 2004; Hiromura et al, 2018). This is notable due to diverse activation pathways associated with CD26-caveolin-1-mediated signaling in different types of immune cells. On one hand, in T cells, ligation of mDPP-4 by caveolin-1 recruits CARMA1 and induces T-cell proliferation and NF-*κ*B activation in a TCR-CD3-dependent manner (Ohnuma et al, 2007). On the other hand, in APCs, binding to mDPP-4 leads to disengagement from caveolin-1 of Toll-interacting protein (Tollip) and interleukin-1 receptor-associated serine/threonine kinase 1 (IRAK-1) with subsequent IRAK-1-induced upregulation of CD86 expression, thereby mobilizing the immune response (Ohnuma et al, 2004). A blockade of the mDPP-4–caveolin-1 signaling axis by fusion protein caveolin-1-Ig leads to anergy in CD4+ T-cells (Ohnuma et al, 2009).

### D GABA-induced signaling in islet endocrine and immune cells

#### 1 Restoring paracrine crosstalk in the islet through facilitating GABAergic transmission in the islets

Menegaz and colleagues were able to establish that the islets of patients with T1D exhibit a significant reduction in GABA levels, which is not caused by a deficiency in GAD65 expression in *β*-cells, suggesting that loss of GABAergic signaling mechanisms may strongly correlate with T1D pathogenesis (Menegaz et al, 2019). They have, for the first time, elegantly identified the exact mechanisms of GABA release from the cytosol via volume regulatory anion channels (VRAC) in a pulsatile fashion independent of glucose concentration (Fig. 1) (Menegaz et al, 2019). As mentioned above, GABAergic transmission is a key *β*-cell mechanism for glucagon regulation and suppression. As GABA is continuously secreted by the *β*-cell, upon GSIS, the secreted insulin initiates downstream signaling through IR in the *α*-cells, leading to PI3K-AKT regulated assembly and translocation of GABAA receptors to *α*-cell membrane. GABAA receptors are chloride channels; once activated by extracellular available GABA, they allow chloride influx and hyperpolarize *α*-cell membrane shutting voltage-gated calcium channels, thereby inhibiting glucagon release (Fig. 3) (Xu et al, 2006). Other groups have also demonstrated protective and regenerative properties of GABA on the *β*-cells (Tian et al, 2013, 2017; Purwana et al, 2014). A recent study using a combination of orally administered GABA with alum-formulated human recombinant GAD65 (GAD65 Alum) in newly diagnosed T1D pediatric patients (GABA: 41 patients, GABA+GAD65 Alum: 25 patients, placebo: 31 patients) showed a significant reduction in fasting and meal-stimulated glucagon levels compared with control (Martin et al, 2022). The authors noted a high safety profile and good tolerability of GABA administration in exclusively pediatric patients and speculated on suitability for higher dosages for increased drug efficacy in future studies.

#### 2 GABAergic signaling in immune cells

Dionisio and colleagues describe a comprehensive GABAergic system in lymphocytes that regulates activation and stimulus responses in T-cells (Dionisio et al, 2011). GABA uptake by the cells is directly dependent on receptor activation, which may be caused by ambient GABA gradients in physiological compartments or by secreted GABA by an autocrine or paracrine loop. Earlier studies also show that 100nM and higher concentrations of GABA inhibit T-cell proliferation and have an immunomodulatory effect by means of interaction with GABAA channels (Bjurstöm et al, 2008). More recent research on antitumor immunity that can be considered for autoimmune conditions also shows that activated B-cells secrete GABA, which directly promotes monocyte differentiation into IL-10-producing anti-inflammatory macrophages that inhibit CD8+ T-cell killer function (Zhang et al, 2021). Bhandage and colleagues have conducted an extensive investigation on the role of GABA in regulating immune cells in relation to T1D. They found that GABA successfully inhibited 16 of 26 cytokines that were significantly elevated in the plasma of T1D patients, as observed in cell assays (Bhandage et al, 2018). Importantly, the authors note that GABAA receptors in the brain seldom contain the ρ2 subunit, which is most prevalent in human peripheral blood mononuclear cells (PBMCs) presenting a specific therapeutic target for autoimmune diseases such as T1D. This research on T cells from T1D patients also reveals that depending on concentration, GABA regulates the release of up to 47 cytokines from human PBMCs and CD4+ T cells (Fig. 4). This aligns with previous research that has shown the inhibitory effects of GABA on several cytokines commonly associated with T1D manifestation and progression, such as IL-2 (Tian et al, 2004), interferon gamma (Tian et al, 2004), IL-6, IL-12 (Reyes-García et al, 2019), IL-1*β* (Zhao et al, 2014), and TNF-*α* (Swaroop et al, 2012).

Under these conditions, identifying novel applications of extrasynaptic GABAA and GABAB receptor modulators in autoimmune disease might be beneficial for investigating the impact of GABAergic signaling on inflammation and the control of immune cells. Treatment with lesogaberan, a peripherally restricted GABAB receptor agonist alone and in combination with anti-CD3 increased remission and rapidly inhibited *β*-cell destruction in NOD mice (Tian et al, 2021). In anti-CD3-stimulated human PBMCs, GABAAR was found to be responsible for inhibiting Ca2+ influx and NF-kB transcriptional activity (Alam et al, 2006; Prud’homme et al, 2013). As an example, diazepam, a positive allosteric modulator of GABAA receptors, successfully inhibited interferon gamma production in anti-CD3 stimulated human and murine CD4+ and CD8+ T cells (Wei et al, 2010; Soltani et al, 2011) and was shown to inhibit innate and adaptive immune responses in a number of animal studies (Sanders et al, 2015; Falcón et al, 2021). Evidently, GABA signaling plays an important role in regulating immune cell behaviors and cytokine release. As research results on GABA efficacy in T1D remain inconsistent, it is possible that other molecular pathways need to be activated for GABA to be sufficiently produced by pancreatic cells and subsequently reabsorbed by the immune cells in conditions of inflammation, cytokine assault, and glucotoxicity, associated with T1D.

## VII Combination of pharmaceutical compounds simultaneously targeting *β*-cells and immune cells

### A Combination of GABA and GAD65 Alum

A study we referenced earlier (Martin et al, 2022) included a treatment arm with 2-dose regimen of GAD65 Alum in combination with twice-daily oral GABA in 25 recent-onset T1D pediatric patients. Further assessment of PBMCs of patient cohorts from this study by Heath and colleagues established that GABA and GAD65 Alum combination treatment showed a significant upregulation of IL-21 and inflammatory CXCL10 at 5 months posttreatment (*P* < .05; Heath et al, 2023). Notably, GABA, by itself, did not have such an effect. FOXP3 transcription factor, which functions as the primary regulator of Treg differentiation, showed a significant increase after 12 months of GABA intervention. However, there was no such increase seen in the GABA with the GAD65 Alum group. Both GABA and GABA with GAD65 Alum showed some inhibition of TNF-*α* and IFN-*γ* synthesis in Th1, the latter possibly by downregulating IL-12p40 promoter expression 5–12 months posttreatment. The combination of GABA and GAD65 Alum retained this effect also at 12 months posttreatment (*P* < .05).

Importantly, the authors assessed differences in response to GABA and GAD65 Alum treatment from patients with T1D-associated HLA-DR3-DQ2 and HLA-DR4-DQ8 haplotypes (both HLA class II). They established that HLA-DR3 patients responded with significant downregulation of CXCL10 (*P* < .05) to a combination of GABA with GAD65 Alum, and downregulation of IFN-*γ* to both GABA as well as GABA with GAD65 Alum intervention, compared with both placebo (*P* < .001) and HLA-DR4 patients (*P* < .001). These findings are consistent with a previously reported clinical study identifying HLA-DR3 patients as better responders to vitamin D and GAD65 Alum combination treatment (Ludvigsson et al, 2021b) and research observing the positive dose-dependent response to GAD65 Alum in HLA-DR3 positive patients, but not in HLA-DR4 haplotype carriers (Hannelius et al, 2020).

Together, these findings are especially valuable in showing the importance of identifying genetic predisposition before commencing specific interventions to achieve the best therapeutic outcomes. HLA-DR3 haplotype in recent-onset T1D patients seems to be especially responsive to GAD65 Alum and GABA interventions. Collectively, there is evidence that both GABA and GAD65 Alum interventions can alter the phenotype of Teff cells and inhibit the synthesis of some proinflammatory cytokines and chemokines from PBMCs of T1D patients. Despite this, several studies also point out that administration of GAD65 Alum intervention in stage 3 T1D patients contributes to the elevation of several inflammatory markers and cytokines, particularly IL-21 and IL-17, which play substantial roles in autoimmune assault of the *β*-cells, immune cell priming, and antigen secretion from B-cells. This particular effect of GAD65 Alum in immune cells could explain the failure to preserve C-peptide in several clinical studies in recent-onset T1D patients. Notwithstanding the above, a high safety profile of GAD65 Alum, T-cell phenotype alteration, and its promising effect in stage 1 T1D patients can yet make it an effective agent for preventative and combinatory treatments in prediagnosed antibody-positive high-risk groups.

### B Combination of anti-IL-21 and GLP-1RA

IL-21 is primarily produced by CD4+ Tfh cells, with smaller synthesis by Th1 and Th17 cells (Chtanova et al, 2004; Bryant et al, 2007). It is the key cytokine for the activation, proliferation, and trafficking of B lymphocytes into the pancreatic islets (Van Belle et al, 2012) and inducing their differentiation into antigen-producing plasma cells (Ettinger et al, 2005). IL-21 has been widely recognized to promote the progression of T1D in humans (Asano et al, 2007; Ferreira et al, 2015). An elegant clinical study on 553 adults with recent-onset T1D combined mild immunosuppression in the form of anti-IL-21 blockade with GLP-1RA (liraglutide) aimed at improving *β*-cell function and preventing apoptosis (von Herrath et al, 2021). The patients were split into 3 treatment arms and placebo. The combination treatment (77 patients) showed a significant delay of MMTT-stimulated C-peptide depletion (*P* = .0017) compared with placebo, whereas treatment with individual GLP-1RA and anti-IL-21 did not achieve efficacy. In the meantime, the observed HbA1c decrease in all treatment arms did not reach statistical significance, and the combination treatment was well tolerated, with the majority of adverse events associated with gastrointestinal disorders, a known effect of GLP-1RA. This study is of particular interest as a major undertaking to provide a complex disease-modifying treatment, targeting both autoimmune and metabolic dysfunctions with a combination of immune therapy and a *β*-cell-focused agent with possible immunomodulation and anti-inflammatory properties (Ganor et al, 2007; Chiou et al, 2019; Moschovaki Filippidou et al, 2020; Song et al, 2022).

### C Combination of vitamin D with DPP-4i

From an immunomodulatory perspective, there are important effects of vitamin D treatment to consider, including low toxicity and high safety profiles, which make it a compelling candidate for a variety of combination treatments. As we mentioned earlier, vitamin D was a part of several combinations with GAD65 Alum (Ludvigsson et al, 2021b), including additional etanercept (Ludvigsson et al, 2021a) and ibuprofen (Ludvigsson et al, 2020). A combination of vitamin D with DPP-4i is discussed by Pinheiro and colleagues in an individual case report on 2 recent-onset T1D patients aged 20 and 21 years old (Pinheiro et al, 2016) and a retrospective analysis on 46 recent-onset T1D patients (treatment: 27 patients and control: 19 patients; Pinheiro et al, 2023) and report significant extension of partial remission, also known as the “honeymoon phase.” This is an interesting combination approach to scalable immunomodulation with high safety profiles of both components, with the added value of metabolic and immune targets accessible to DPP-4i that can be complemented by broad-spectrum cellular mechanisms addressed by vitamin D.

### D Combination of DPP-4i and PPI

Combination therapy with GLP-1 and gastrin restored normoglycemia in NOD mice with autoimmune diabetes by downregulating autoimmunity and restoring pancreatic islet *β*-cell mass and insulin secretion (Suarez-Pinzon et al, 2008). Diabetes reversal in NOD mice could also be achieved by raising blood levels of endogenous GLP-1 and gastrin, using a DPP-4i and a PPI combination therapy (Suarez-Pinzon et al, 2009). In addition, DPP-4i and PPI combination therapy induced human *β*-cell neogenesis by transdifferentiation from adult human pancreatic exocrine duct cells (Suarez-Pinzon and Rabinovitch, 2011). The REPAIR-T1D phase 2 clinical trial assessed the combination DPP-4i and PPI in 68 recent-onset T1D patients aged 11–36 years (treatment: 46 patients, placebo 22 patients; Griffin et al, 2014). At 12 months, preservation of *β*-cell function as shown by 2-hour AUC C-peptide, insulin demand, and HbA1c levels was not significantly different in active treatment and placebo treatment groups. The combination therapy was well tolerated with a high safety profile, and no adverse events were recorded with respect to the treatment. The authors note that patient response to sitagliptin and lansoprazole was heterogeneous, responding patients had an elevation in GLP-1 and gastrin that was associated with a trend toward improvement in C-peptide, whereas nonresponders did not. Intriguingly, a comprehensive review of drug-drug interactions of different PPIs with metformin or DPP-4i show that lansoprazole and rabeprazole have a weaker potential for interaction with sitagliptin and other DPP-4i when compared with omeprazole, which has a high affinity with several CYP enzymes, possibly significantly affecting overall combination efficacy (Tasnim et al, 2024).

It is worth noting that PPIs, like omeprazole, may also play a role in immune regulation in individuals with type 1 diabetes (T1D). In vitro studies show that PPI can significantly downregulate interferon gamma and IL-2 cytokine expression in PBMCs via blocking lysosomal Zrt-/Irt-like protein zinc transporter (Zip8), thereby effectively shifting the distribution of intracellular zinc (Liu et al, 2023). Studies in other inflammatory diseases, such as idiopathic pulmonary fibrosis, show that esomeprazole can suppress a range of proinflammatory molecules, including TNF-*α*, IL-1*β*, and IL-6 (Ghebremariam et al, 2015; Ghebre, 2018; Pham et al, 2019), which play important roles in T1D pathogenesis. More studies are needed to assess the individual target efficacy of esomeprazole in the context of T1D autoimmune assault; however, large available clinical safety data and oral route of administration make it a promising candidate for combination treatments.

### E Combination of GABA, DPP-4i, and PPI

Our own small retrospective study in 19 T1D adult patients examined the combination of GABA, DPP-4i, and omeprazole (recent-onset T1D: 10 patients, advanced T1D: 9 patients). We compared clinical parameters between baseline and at 26–42 weeks of treatment, and intriguingly, in recent-onset T1D patients, we observed a significant increase in the fasting C-peptide level (*P* = .013) and a significant decrease in HbA1c (*P* = .003), fasting blood glucose (*P* = .033), and insulin/weight ratio (*P* = .028). Advanced T1D patients showed significant improvement in fasting blood glucose (*P* = .021) and insulin dose-adjusted HbA1c (*P* = .031), but not in C-peptide and HbA1c levels (Rabinovitch et al, 2023). These findings on the efficacy of the 3-drug combination of GABA, a DPP-4i, and a PPI in patients with T1D compared with the ineffectiveness of the 2-drug combination of a DPP-4i and a PPI in the REPAIR-T1D trial is consistent with our earlier findings in NOD mice that diabetes could be reversed by the 3-drug combination of GABA, a DPP-4i, and a PPI, but not by any of the different 2-drug combinations of these 3 drugs (Lagunas-Rangel et al, 2022). GABA may have therapeutic effects on T1D via several mechanisms of action. First, GABA increases the secretion of GLP-1 (Gameiro et al, 2005) and gastrin (Weigert et al, 1998). These actions would add to the insufficient increases in GLP-1 and gastrin in response to combination therapy with DPP-4i and PPI in the REPAIR-T1D trial. In addition, GABA induces signaling in islet endocrine cells and in immune cells, both of which actions would be therapeutic in T1D, as discussed earlier (see section GABA-induced signaling in islet endocrine and immune cells).

## VIII Conclusion

The multifactorial nature of T1D and its inherent heterogeneity ensures that response to every treatment depends on many factors, including, but not limited to age, genetic predisposition of *β*-cell fragility, high-risk HLA complex expression, and particular susceptibility to viral triggers (see section Initiation and progression of type 1 diabetes). This significantly complicates a decade-long search for a universal T1D curative drug that can promote sustainable remission.

This review supports previous works identifying T1D as a condition encompassing dual vulnerability of immune and endocrine systems. For instance, *β*-cell function, insulin secretion, and paracrine signaling system are entirely reliant on a delicate balance of glucose, hormones, incretins, and neurotransmitters regulating voltage potential of cell membranes through various neuron-like ion channels and transporters (see Fig. 1 and section Appropriate β-cell function and regulation of electrical activity). Figures 2 and 3 clearly show that a single major dysfunction like prolonged hyperglycemia can initiate a number of downward proapoptotic signaling cascades in a domino-like effect, capable of engaging several molecular pathways at a time. In the meantime, autoimmune response in T1D is also not a singular linear event, with the majority of activation mechanisms still not fully elucidated. As such, viral triggers as well as metabolic dysfunctions can facilitate significant inflammatory *β*-cell damage and secretion of hybrid peptides and other molecules, thereby contributing to the spreading of autoantigens to be processed by APCs and initiation of autoimmunity (see section Interaction of genetic and environmental factors in type 1 diabetes).

There are also several distinct identifiable T-cell activation pathways involving dynamic antigen presentation by HLA class I and class II complexes. The latter is specific to T1D-associated HLA-DR, -DQ haplotypes that allow for distressed *β*-cells to directly engage islet-infiltrating CD4+ T cells in an APC-like manner (see Fig. 2 and section Human leukocyte antigen genetic risk factors associated with type 1 diabetes). Importantly, T-cell activation by itself is a complex process, with costimulatory receptors (eg, CD28 and CD26) and coinhibitory receptors (eg, CTLA-4, PD-1, and TIGIT) determining the functional outcome of primary TCR-CD3 stimulation. Given the multitude of autoimmune targets that emerge as T1D progresses, the potential for immune interventions is extensive but remains constrained by safety concerns and compensatory mechanisms that can effectively circumvent targeted immunosuppression. Some of the reviewed interventions encompass a wide range of approaches, including the use of broad immunosuppressants to exhaust and alter T-cell phenotype (see Fig. 4 and section Pharmaceutically induced T-cell blockade leading to partial or full exhaustion), the inhibition of specific cytokines associated with T1D progression (see section Pharmaceutical inhibition of cytokines and corresponding receptors), and the modulation of immunity by enhancing immunologic tolerance (see section Modifying immunity through autoantigens: GAD65 Alum).

Some of the reviewed pathways initiated by hyperglycemia coincide with mechanistic targets of successful pharmaceutical interventions, such as TxNIP assembly that was seen to be mechanistically inhibited by verapamil through the calcium influx blockade (see Fig. 4 and section Calcium-induced inhibition of TxNIP assembly in β-cells), and ER stress directly targeted by imatinib through inhibition of cytosolic ABL kinase transport (see Fig. 4 and section Imatinib-induced mediation of β-cell ER stress). To provide a thorough understanding of addressable intervention checkpoints, we have incorporated mechanistic findings from multiple preclinical investigations with detailed explanations of their specific molecular targets. Some of them can mediate metabolic dysfunction by reducing glutamate-induced *β*-cell damage, improving GSIS, and stabilizing calcium oscillations via NMDAR blockade with DXM (see section Glutamate-induced β-cell damage) and rescuing *β*-cells by attenuating TxNIP expression and inducing cAMP-PKA-*β*-catenin survival pathway with a combination of GABA and GLP-1RA (see Fig. 3 and section Wnt/β-Catenin pathway-induced β-cell proliferation and survival).

Looking at extensive available research, we also find that utilizing *β*-cell capacity for survival under stress can be addressed by several drugs initiating signaling through different GPCRs, depending on the desired effect. As such, GLP-1RA or DPP-4i induced cAMP-PKA/Epac2 signaling can potentiate GSIS and upregulate PDX1, whereas multiple binding ligands to several G_i/o_-GPCRs can initiate PI3K-AKT-mTORC1 downstream effector signaling for cellular metabolism and survival under stress (see Fig. 3 and sections Pancreatic β-cells regulation in health and disease and targeting β-cell homeostasis through G protein-coupled receptors). Although clinical results show that targeting these individual pathways was not sufficient to facilitate meaningful clinical efficacy alone, using them to alleviate *β*-cell stress in combination with complementary components or even immune therapy can be effective, as seen by combining GLP-1RA with anti-IL-21 (see section Combination of Anti-IL-21 and GLP-1RA). Some clinical studies especially reflect on the unpredictability of possible outcomes in combinatorial treatments. An example of this would be the combination of GAD65 Alum with vitamin D and etanercept that led to a significant upregulation of TNF-*α* among other T1D-associated cytokines (see section Combining vitamin D with GAD65 Alum and etanercept). Nevertheless, by assessing available interventions collectively, we were able to elucidate which individual immune therapies have a transient effect on C-peptide preservation, evaluate their clinical safety, and contextualize these findings within the framework of established disease pathophysiology (see section Combination of pharmaceutical compounds simultaneously targeting β-cells and immune cells).

In this review, we have described those molecular mechanisms reported to be involved in the pathogenesis of T1D. The clinical trials of various interventions described in this review were designed to interrupt these mechanisms of disease. Limitations or ineffectiveness of these therapeutic interventions may reflect incomplete or irrelevant information on mechanisms thought to be operative in the pathogenesis of T1D. The challenge in translating mechanistic insights into effective treatments might be to uncover more complete and relevant information on those mechanisms contributing to the development of T1D.

The experimental animal models of T1D described in this review should not be considered perfect models of human T1D but rather of a form of diabetes particular to that animal. For example, the autoimmune response against islet *β*-cells in the NOD mouse, the most used animal model, is unlikely to be a perfect representation of the autoimmune response in humans with T1D, in whom this response is more likely to have multiple pathways. Mechanisms of disease would be highly uniform in an inbred animal such as the NOD mouse, but variable in an outbred human population. Indeed, this may explain why many “cures” of T1D reported for NOD mice have not succeeded in patients with T1D. One potential area to improve the relevance of preclinical studies would be that of experimental design. We would suggest designs where potential disease-modifying therapies are added as adjuncts to insulin therapy only 2–4 weeks after diabetes diagnosis in the animal models in order to replicate more closely the situation in human trials of these therapies.

As discussed in our review, the main benefit of a combinatorial approach to therapy is the opportunity to simultaneously address multiple autoimmune and metabolic dysfunctions of islet *β*-cells in T1D. However, challenges exist in the clinical development of the combination therapy approach emanating from both the clinical and regulatory implementation. From a clinical perspective, when a single therapeutic drug is used, there is only the route of administration and dosing regimen of one component to consider. In the combination approach, the patient may be presented with different oral and parenteral dosage forms, and these may not have the convenience of coadministration. This complexity may influence the recruitment of patients, prolonging study time. Furthermore, great care must be taken to ensure that the patient complies with a complicated dosage regimen. Furthermore, in order to authorize a combination therapy for marketing, regulators need to be convinced that using multiple drugs in combination is safe and clinically superior to any of the components used alone or any other subset of the drugs being proposed in combination. Even considering the use of factorial design, this could lead to the need for multiarm clinical studies comparing singles, double combinations, and more, vastly adding to the number of subjects required, and as a result in the time, complexity, and cost of the study. In this context, the enthusiasm for novel research opportunities and efforts directed at assessing synergies of combinatorial treatments must be carefully balanced with the realities of clinical applicability and regulatory frameworks, which have the potential to both facilitate the search for a curative intervention and irrevocably halt it.

## Conflict of interest

Shmuel Levit, Daniil Koshelev, and Liudmila Kosheleva are members of Levicure LTD and have patents related to the triple combination of GABA, DPP-4i, and PPI. The remaining authors declare that the research was conducted in the absence of any commercial or financial relationships that could be construed as a potential conflict of interest.
